# Antimicrobial and ADME properties of methoxylated, methylated and nitrated 2-hydroxynaphthalene-1 carboxanilides

**DOI:** 10.5599/admet.2642

**Published:** 2025-02-08

**Authors:** Lucia Vrablova, Tomas Gonec, Tereza Kauerova, Michal Oravec, Izabela Jendrzejewska, Peter Kollar, Alois Cizek, Josef Jampilek

**Affiliations:** 1 Department of Analytical Chemistry, Faculty of Natural Sciences, Comenius University, Ilkovicova 6, 842 15 Bratislava, Slovakia; 2 Department of Chemical Drugs, Faculty of Pharmacy, Masaryk University, Palackeho tr. 1946/1, 612 00 Brno, Czech Republic; 3 Department of Pharmacology and Toxicology, Faculty of Pharmacy, Masaryk University, Palackeho tr. 1946/1, 612 00 Brno, Czech Republic; 4 Global Change Research Institute CAS, Belidla 986/4a, 603 00 Brno, Czech Republic; 5 Institute of Chemistry, University of Silesia, Bankowa 12, 40007 Katowice, Poland; 6 Department of Infectious Diseases and Microbiology, Faculty of Veterinary Medicine, University of Veterinary Sciences Brno, Palackeho tr. 1946/1, 612 42 Brno, Czech Republic; 7 Department of Chemical Biology, Faculty of Science, Palacky University Olomouc, Slechtitelu 27, 779 00 Olomouc, Czech Republic

**Keywords:** Lipophilicity, antibacterial activity, antimycobacterial activity, cytotoxicity

## Abstract

**Background and purpose:**

Many new compounds are being prepared to overcome the problem of increasing microbial resistance and the increasing number of infections.

**Experimental approach:**

This study includes a series of twenty-seven mono-, di- and trisubstituted 2-hydroxynaphthalene-1-carboxanilides designed as multitarget agents. The compounds are substituted with methoxy, methyl, and nitro groups, as well as additionally with chlorine, bromine, and trifluoromethyl at various positions. All the compounds were evaluated for antibacterial activities against Gram-positive and Gram-negative bacteria and mycobacteria. Cytotoxicity on human cells was also tested.

**Key results:**

Three compounds showed activity comparable to clinically used drugs. *N*-(3,5-Dimethylphenyl)-2-hydroxynaphthalene-1-carboxamide (**13**) showed only antistaphylococcal activity (minimum inhibitory concentration (MIC) = 54.9 μM); 2-hydroxy-*N*-[2-methyl-5-(trifluoromethyl)phenyl]naphthalene-1-carboxamide (**22**) and 2-hydroxy-*N*-[4-nitro-3-(trifluoromethyl)phenyl]naphthalene-1-carboxamide (**27**) were active across the entire spectrum of tested bacteria/mycobacteria, both against the sensitive set and against resistant isolates (MICs range 0.3 to 92.6 μM). Compound **22** was even active against E. coli (MIC = 23.2 μM). The active agents showed no *in vitro* cytotoxicity up to a concentration of 30 μM.

**Conclusion:**

Compounds with trifluoromethyl in the *meta*-anilide position, experimental lipophilicity expressed as log *k* (logarithm of the capacity factor) in the range of 0.31 to 0.34 and calculated electron σ parameter for the anilide substituent higher than 0.59 were effective. The investigated compounds meet the definition of Michael acceptors. Based on ADME screening, the investigated compounds **13**, **22** and **27** should have suitable physicochemical parameters for good bioavailability in the organism. Therefore, these are promising agents for further study.

## Introduction

An increasingly common problem is the increasing number of infections caused by a wide range of microorganisms. The increase in antimicrobial resistance across the spectrum of bacteria plays a major role [1-3]. The most effective tool to counter this unfortunate trend is the effort to discover new bioactive compounds or at least innovated structures of existing drugs with a new/innovative mechanism of action [4-7]. In addition to the development of the most valuable molecules – structurally novel anti-infectives targeting new (single or multiple) targets [8,9], interesting strategies include the development of lantibiotics and bacteriocins [10,11], antimicrobial peptides and bacterial cell membrane disruptors [12-15], chemosensitizers, inhibitors of *quorum sensing*, virulence and biofilm formation, phage or monoclonal antibody-based therapies [16-18], drug repurposing [19,20], or nanoparticle-based strategies [21,22].

As mentioned, the current trend remains the design of so-called multitarget compounds, which are able to act on many different targets and thus interfere with the bacterial microorganism at different points of metabolism or reproduction [18,23-27]. Salicylanilides represent a promising type of multitarget compounds [28-36]. Inspired by salicylanilides, deeper research into their cyclic analogues – hydroxynaphthalenecarboxanilides – was initiated. These compounds are characterized not only by antimicrobial [37] but also by antiparasitic [38] and anticancer [39] activity. In these compounds, the essential role of the hydroxyl group has been identified, which must be free [40,41] or substituted by a group (*e.g.* carbamate [42]) capable of forming bonds with biomolecules. The connecting amide bridge between aromatic systems is also an important part. The position of the phenyl group is important because it manifests various physicochemical (*e.g.* solubility and lipophilicity), but also biological properties [37-39,43-45]. Overall, it can be said that the mentioned molecules can be considered as Michael acceptors [46-51].

Many anilides have been prepared, especially with pronounced lipophilic and electron-withdrawing (F/Cl/Br, CF_3_) substituents, which were expected to be antimicrobially active, *i.e.* the molecules would approach the properties of Michael acceptors [37,38,40,41,43-45]. Only a few works have dealt with alkoxy or methyl substituents [52-54]. Thus, it is a follow-up work of previous research, where anilides containing various combinations of predominantly polar and electron-donating/less electron-accepting substituents on the 2-hydroxynaphthalene-1-carboxanilide scaffold have now been synthesized and all the prepared compounds have been investigated on a wide battery of bacterial and mycobacterial species.

## Experimental

### General

All reagents were purchased from Merck (Sigma-Aldrich, St. Louis, MO, USA) and Alfa (Alfa-Aesar, Ward Hill, MA, USA). Microwave-assisted reactions were performed using a StartSYNTH microwave lab station (Milestone, Sorisole BG, Italy). The melting points were determined on a Kofler hot-plate apparatus HMK (Franz Kustner Nacht KG, Dresden, Germany) and were uncorrected. Infrared (IR) spectra were recorded on an ATR diamond iD7 for Nicolet™ Impact 410 Fourier-transform IR spectrometer (Thermo Scientific, West Palm Beach, FL, USA). The spectra were obtained by accumulating 64 scans with a 2 cm^–1^ resolution in the region of 4000-650 cm^–1^. All ^1^H- and ^13^C-NMR spectra were recorded on a JEOL ECZR 400 MHz NMR spectrometer (400 MHz for ^1^H and 100 MHz for ^13^C, Jeol, Tokyo, Japan) in dimethyl sulfoxide-*d*_6_ (DMSO-*d*_6_). ^1^H and ^13^C chemical shifts (*ẟ*) are reported in ppm. High-resolution mass spectra were measured using a high-performance liquid chromatograph Dionex UltiMate® 3000 (Thermo Scientific, West Palm Beach, FL, USA) coupled with an LTQ Orbitrap XL™ Hybrid Ion Trap-Orbitrap Fourier Transform Mass Spectrometer (Thermo Scientific) equipped with a HESI II (heated electrospray ionization) source in the positive mode.

### General procedure for synthesis of N-(substituted phenyl)-2-hydroxynaphthalene-1-carboxamides **1-27**.

2-Hydroxynaphthalene-1-carboxylic acid (5.3 mmol) and the corresponding substituted aniline (5.3 mmol) were suspended in 30 mL of dry chlorobenzene. Phosphorous trichloride (2.65 mmol) was added dropwise, and the reacting mixture was heated in the microwave reactor for 15 min at 130 °C and maximal allowed power 500 W using infrared flask-surface control of temperature. The solvent was evaporated under reduced pressure, the solid residue was washed with 2 M HCl, and the crude product was recrystallized from aqueous ethanol. All the studied compounds are presented in Table 1.

**Table 1.** Structure of ring-substituted 2-hydroxynaphthalene-1-carboxanilides **1**-**27**; experimentally determined logarithm of the capacity factor (log *k*), logarithm of distribution coefficients at pH 6.5 (log *D*_6.5_) and pH 7.4 (log *D*_7.4_), predicted lipophilicity (log *P*) values and electronic *σ*_(Ar)_ parameters of anilide ring of investigated compounds

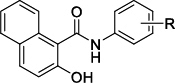						
Comp.	R	log *k*	log *D*_6.5_	log *D*_7.4_	log *P*^1^	*σ*_(Ar)_^1^
**1**	H	0.0340	0.0011	0.0107	4.49	0.60
**2**	2-OCH_3_	0.3181	0.3015	0.3069	4.54	0.01
**3**	3-OCH_3_	0.1751	0.1643	0.1717	4.51	0.66
**4**	4-OCH_3_	-0.0380	-0.0491	-0.0382	4.30	0.36
**5**	2,5-OCH_3_	0.3353	0.3263	0.3310	4.70	0.08
**6**	3,5-OCH_3_	0.0388	0.0411	0.0490	4.28	0.93
**7**	3,4,5-OCH_3_	-0.1150	-0.1053	-0.0948	4.22	0.69
**8**	2-CH_3_	0.0858	0.0745	0.0838	4.83	0.59
**9**	3-CH_3_	0.1762	0.1644	0.1727	4.83	0.48
**10**	4-CH_3_	0.1658	0.1553	0.1632	4.83	0.46
**11**	2,5-CH_3_	0.2486	0.2455	0.2514	4.99	0.59
**12**	2,6-CH_3_	0.0785	0.0838	0.0923	4.99	0.58
**13**	3,5-CH_3_	0.3367	0.3429	0.3480	4.99	0.59
**14**	2,4,6-CH_3_	0.2539	0.2629	0.2691	4.84	0.44
**15**	2-OCH_3_-5-CH_3_	0.4932	0.4974	0.5012	4.77	0.01
**16**	2-OCH_3_-6-CH_3_	0.0124	0.0284	0.0382	4.77	0.01
**17**	2-CH_3_-5-OCH_3_	0.0892	0.1047	0.1113	4.95	0.76
**18**	2-Cl-5-OCH_3_	0.4635	0.4607	0.4593	5.15	1.13
**19**	2-OCH_3_-5-Br	0.5845	0.5836	0.5835	5.58	0.12
**20**	2-OCH_3_-5-CF_3_	0.5335	0.5323	0.5305	5.77	0.11
**21**	3-CF_3_-4-OCH_3_	0.1635	0.1704	0.1745	5.64	0.58
**22**	2-CH_3_-5-CF_3_	0.3373	0.3444	0.3440	5.71	0.82
**23**	3-CF_3_-4-CH_3_	0.4605	0.4621	0.4636	5.71	0.68
**24**	2-NO_2_	0.3033	0.3077	0.3103	4.45	1.12
**25**	3-NO_2_	0.4882	0.3462	0.3058	4.50	1.09
**26**	4-NO_2_	0.0984	0.0862	0.0879	4.59	1.14
**27**	3-CF_3_-4-NO_2_	0.3124	0.3282	0.3220	5.47	1.36

^1^calculated using ACD/Percepta ver. 2012 (Advanced Chemistry Development, Inc., Toronto, ON, Canada, 2012) [55].

2-Hydroxy-*N*-phenylnaphthalene-1-carboxamide (**1**), 2-hydroxy-*N*-(2-methoxyphenyl)naphthalene-1-carboxamide (**2**), 2-hydroxy-*N*-(3-methoxyphenyl)naphthalene-1-carboxamide (**3**), 2-hydroxy-*N*-(4-methoxyphenyl)naphthalene-1-carboxamide (**4**), 2-hydroxy-*N*-(2-methylphenyl)naphthalene-1-carboxamide (**8**), 2-hydroxy-*N*-(3--methylphenyl)naphthalene-1-carboxamide (**9**), 2-hydroxy-*N*-(4-methylphenyl)naphthalene-1-carboxamide (**10**), 2-hydroxy-*N*-(2-nitrophenyl)naphthalene-1-carboxamide (**24**), 2-hydroxy-*N*-(3-nitrophenyl)naphthalene-1-carboxamide (**25**), 2-hydroxy-*N*-(4-nitrophenyl)naphthalene-1-carboxamide (**26**) were described by Gonec *et al.* [43].

#### *N*-(2,5-Dimethoxyphenyl)-2-hydroxynaphthalene-1-carboxamide (5)

Yield 77 %; mp 196-198 °C; IR (cm^–1^): 2934, 2829, 1625, 1583, 1531, 1513, 1486, 1463, 1434, 1423, 1353, 1278, 1265, 1239, 1219, 1175, 1148, 1033, 970, 959, 895, 847, 815, 792, 741, 717, 702; ^1^H-NMR (DMSO-*d_6_*), *ẟ*: 10.50 (br. s, 1H), 9.32 (s, 1H), 8.06 (d, 1H, *J*=2.7 Hz), 8.02 (d, 1H, *J*=8.7 Hz), 7.88 (d, 1H, *J*=9.1 Hz), 7.84 (d, 1H, *J*=7.8 Hz), 7.48 (ddd, 1H, *J*=8.2 Hz, *J*=6.9 Hz, *J*=0.9 Hz), 7.32-7.36 (m, 1H), 7.25 (d, 1H, *J*=9.1 Hz), 7.00 (d, 1H, *J*=8.7 Hz), 6.68 (dd, 1H, *J*=8.7 Hz, *J*=3.2 Hz), 3.77 (s, 3H), 3.76 (s, 3H) (see Figure S1 in Supplementary Materials); ^13^C-NMR (DMSO-*d_6_*), *ẟ*: 165.31, 153.13, 152.34, 143.23, 131.76, 131.13, 128.52, 128.04, 127.72, 127.07, 123.96, 123.12, 118.27, 116.77, 111.99, 108.05, 107.47, 56.40, 55.44 (Figure S2); HR-MS: [M+H]^+^ calculated 324.123034 *m*/*z*, found 324.12305 *m*/*z*.

#### *N*-(3,5-Dimethoxyphenyl)-2-hydroxynaphthalene-1-carboxamide (**6**)

Yield 64 %; mp 151-153 °C; IR (cm^–1^): 3266, 2999, 2934, 2833, 2540, 1614, 1595, 1549, 1514, 1470, 1453, 1423, 1332, 1296, 1257, 1227, 1194, 1154, 1064, 985, 846, 813, 739, 711; ^1^H-NMR (DMSO-*d_6_*), *ẟ*: 10.31 (s, 1H), 10.12 (s, 1H), 7.86 (d, 2H, *J*=8.4 Hz), 7.67 (d, 1H, *J*=8.4 Hz), 7.47 (td, 1H, *J*=7.3 Hz, *J*=1.1 Hz), 7.33 (td, 1H, *J*=7.3 Hz, *J*=1.1 Hz), 7.25 (d, 1H, *J*=8.8 Hz), 7.09 (s, 2H), 6.27 (s, 1H), 3.74 (s, 6H) (Figure S3); ^13^C-NMR (DMSO-*d_6_*), *ẟ*: 165.83, 160.51, 151.60, 141.30, 131.37, 130.14, 127.96, 127.38, 126.97, 123.38, 123.00, 118.61, 118.34, 97.66, 95.38, 55.08 (Figure S4); HR-MS: [M-H]^+^ calculated 322.10738 *m*/*z*, found 322.10788 *m*/*z*.

#### 2-Hydroxy-*N*-(3,4,5-trimethoxyphenyl)naphthalene-1-carboxamide (**7**)

Yield 43 %; mp 223-225 °C; IR (cm^–1^): 3335, 2943, 2834, 1636, 1586, 1541, 1505, 1448, 1406, 1347, 1299, 1281, 1128, 1030, 1000, 980, 969, 891, 817, 774, 745, 686; ^1^H-NMR (DMSO-*d_6_*), *ẟ*: 10.29 (s, 1H), 10.11 (s, 1H), 7.86 (d, 1H, *J*=8.7 Hz), 7.85 (d, 1H, *J*=8.2 Hz), 7.68 (d, 1H, *J*=8.2 Hz), 7.46 (ddd, 1H, *J*=8.2 Hz, *J*=6.9 Hz, *J*=0.9 Hz), 7.31-7.35 (m, 1H), 7.25 (s, 2H), 7.25 (d, 1H, *J*=8.7 Hz), 3.76 (s, 6H), 3.65 (s, 3H) (Figure S5); ^13^C-NMR (DMSO-*d_6_*), *ẟ*: 165.58, 152.74, 151.62, 135.95, 133.39, 131.41, 130.15, 127.98, 127.38, 126.97, 123.47, 123.02, 118.65, 118.33, 96.86, 60.17, 55.70 (Figure S6); HR-MS: [M+H]^+^ calculated 354.133599 *m*/*z*, found 354.13345 *m*/*z*.

#### *N*-(2,5-Dimethylphenyl)-2-hydroxynaphthalene-1-carboxamide (**11**)

Yield 71 %; mp 162-165 °C; IR (cm^–1^): 2914, 1646, 1576, 1515, 1434, 1406, 1319, 1261, 1141, 1033, 962, 902, 873, 803, 742, 678, 661; ^1^H-NMR (DMSO-*d_6_*), *ẟ*: 10.13 (br. s, 1H), 9.71 (s, 1H), 7.83-7.87 (m, 3H), 7.50 (ddd, 1H, *J*=8.2 Hz, *J*=6.9 Hz, *J*=1.1 Hz), 7.41 (s, 1H), 7.33 (t, 1H, *J*=7.5 Hz), 7.24 (d, 1H, *J*=9.1 Hz), 7.14 (d, 1H, *J*=7.8 Hz), 6.96 (dd, 1H, *J*=7.8 Hz, *J*=1.1 Hz), 2.32 (s, 3H), 2.28 (s, 3H) (Figure S7); ^13^C-NMR (DMSO-*d_6_*), *ẟ*: 165.56, 151.75, 136.22, 134.83, 131.66, 130.09, 130.03, 129.36, 127.94, 127.47, 126.85, 126.29, 126.05, 123.65, 122.92, 118.42, 118.34, 20.63, 17.62 (Figure S8); HR-MS: [M+H]^+^ calculated 292.133205 *m*/*z*, found 292.13290 *m*/*z*.

#### *N*-(2,6-Dimethylphenyl)-2-hydroxynaphthalene-1-carboxamide (**12**)

Yield 84 %; mp 195-197 °C; IR (cm^–1^): 3352, 2939, 2834, 1623, 1604, 1574, 1514, 1458, 1319, 1139, 1033, 970, 906, 821, 796, 756, 726, 717, 702, 661; ^1^H-NMR (DMSO-*d_6_*), *ẟ*: 10.17 (br.s, 1H), 9.67 (s, 1H), 7.89 (d, 1H, *J*=8.2 Hz), 7.84-7.87 (m, 2H), 7.50 (ddd, 1H, *J*=8.2 Hz, *J*=6.9 Hz, *J*=0.9 Hz), 7.33 (t, 1H, *J*=7.3 Hz), 7.25 (d, 1H, *J*=9.1 Hz), 7.12 (s, 3H), 2.39 (s, 6H) (Figure S9); ^13^C-NMR (DMSO-*d_6_*), *ẟ*: 165.44, 151.83, 135.59, 135.27, 131.77, 129.93, 128.01, 127.68, 127.48, 126.81, 126.42, 123.61, 122.88, 118.60, 118.29, 18.66 (Figure S10); HR-MS: [M+H]^+^ calculated 292.133205 *m*/*z*, found 292.13300 *m*/*z*.

#### *N*-(3,5-Dimethylphenyl)-2-hydroxynaphthalene-1-carboxamide (**13**)

Yield 70 %; mp 181-184 °C; IR (cm^–1^): 3339, 3217, 1612, 1577, 1531, 1514, 1435, 1351, 1310, 1275, 1244, 1231, 1210, 1146, 968, 842, 819, 753, 743, 685; ^1^H-NMR (DMSO-*d_6_*), *ẟ*: 10.22 (s, 1H), 10.07 (s, 1H), 7.84 (d, 2H, *J*=8.4 Hz), 7.67 (d, 1H, *J*=8.4 Hz), 7.49 (td, 1H, *J*=7.0 Hz, *J*=1.1 Hz), 7.34 (s, 2H), 7.32 (td, 1H, *J*=7.0 Hz, *J*=1.1 Hz), 7.26 (td, 1H, *J*=9.1 Hz, *J*=1.3 Hz), 7.34 (s, 1H), 2.27 (s, 6H) (Figure S11); ^13^C-NMR (DMSO-*d_6_*), *ẟ*: 165.62, 151.55, 139.48, 137.58, 131.45, 129.99, 127.93, 127.40, 126.88, 124.80, 123.44, 122.94, 118.76, 118.36, 117.13, 21.17 (Figure S12); HR-MS: [M-H]^+^ calculated 290.11756 *m*/*z*, found 290.11829 *m*/*z*.

#### 2-Hydroxy-*N*-(2,4,6-trimethylphenyl)naphthalene-1-carboxamide (**14**)

Yield 72 %; mp 175-177 °C; IR (cm^–1^): 3338, 2944, 2831, 1623, 1602, 1577, 1512, 1460, 1396, 1369, 1319, 1223, 1206, 1155, 1030, 971, 906, 847, 821, 796, 772, 754, 724, 713, 690, 672; ^1^H-NMR (DMSO-*d_6_*), *ẟ*: 10.13 (br.s, 1H), 9.56 (s, 1H), 7.87 (d, 1H, *J*=8.7 Hz), 7.84 (d, 1H, *J*=8.7 Hz), 7.84 (d, 1H, *J*=9.1 Hz), 7.49 (ddd, 1H, *J*=8.2 Hz, *J*=6.9 Hz, *J*=1.4 Hz), 7.33 (ddd, 1H, *J*=8.1 Hz, *J*=7.0 Hz, *J*=0.9 Hz), 7.24 (d, 1H, *J*=9.1 Hz), 6.93 (s, 2H), 2.34 (s, 6H), 2.26 (s, 3H) (Figure S13); ^13^C-NMR (DMSO-*d_6_*), *ẟ*: 165.52, 151.79, 135.32, 135.27, 132.65, 131.77, 129.86, 128.26, 127.99, 127.47, 126.76, 123.64, 122.85, 118.68, 118.29, 20.53, 18.55 (Figure S14); HR-MS: [M+H]^+^ calculated 306.148855 *m*/*z*, found 306.14862 *m*/*z*.

#### 2-Hydroxy-*N*-(2-methoxy-5-methylphenyl)naphthalene-1-carboxamide (**15**)

Yield 73 %; mp 203-205 °C; IR (cm^–1^): 3342, 2945, 2833, 1633, 1585, 1530, 1514, 1482, 1462, 1434, 1370, 1353, 1321, 1300, 1272, 1257, 1210, 1148, 1123, 1030, 969, 896, 868, 817, 799, 750, 719, 687; ^1^H-NMR (DMSO-*d_6_*), *ẟ*: 10.41 (br.s, 1H), 9.26 (s, 1H), 8.14 (s, 1H), 8.01 (d, 1H, *J*=8.7 Hz), 7.87 (d, 1H, *J*=9.1 Hz), 7.84 (d, 1H, *J*=8.2 Hz), 7.48 (ddd, 1H, *J*=8.7 Hz, *J*=6.9 Hz, *J*=1.4 Hz), 7.33 (ddd, 1H, *J*=8.2 Hz, *J*=6.9 Hz, *J*=0.9 Hz), 7.24 (d, 1H, *J*=8.7 Hz), 6.92-6.98 (m, 2H), 3.78 (s, 3H), 2.31 (s, 3H) (Figure S15); ^13^C-NMR (DMSO-*d_6_*), *ẟ*: 165.22, 152.17, 147.34, 131.76, 130.89, 129.22, 128.00, 127.68, 127.39, 126.98, 124.54, 123.96, 123.06, 121.89, 118.28, 117.12, 111.12, 55.92, 20.58 (Figure S16); HR-MS: [M+H]^+^ calculated 308.12812 *m*/*z*, found 308.12820 *m*/*z*.

#### 2-Hydroxy-*N*-(2-methoxy-6-methylphenyl)naphthalene-1-carboxamide (**16**)

Yield 77 %; mp 142-145 °C; IR (cm^–1^): 3325, 2944, 2833, 1625, 1581, 1514, 1472, 1437, 1354, 1305, 1285, 1139, 1120, 1081, 1031, 970, 912, 818, 761, 741, 712; ^1^H-NMR (DMSO-*d_6_*), *ẟ*: 10.04 (br.s, 1H), 9.46 (s, 1H), 8.09 (d, 1H, *J*=7.8 Hz), 7.83 (d, 1H, *J*=9.1 Hz), 7.83 (d, 1H, *J*=7.8 Hz), 7.52 (ddd, 1H, *J*=8.6 Hz, *J*=7.0 Hz, *J*=1.4 Hz), 7.33 (ddd, 1H, *J*=8.2 Hz, *J*=6.9 Hz, *J*=0.9 Hz), 7.23 (d, 1H, *J*=8.7 Hz), 7.20 (t, 1H, *J*=7.8 Hz), 6.94 (d, 1H, *J*=7.8 Hz), 6.89 (d, 1H, *J*=7.3 Hz), 3.86 (s, 3H), 2.35 (s, 3H) (Figure S17); ^13^C-NMR (DMSO-*d_6_*), *ẟ*: 165.77, 155.34, 151.71, 137.06, 131.81, 129.74, 127.74, 127.42, 127.19, 126.58, 125.14, 124.30, 122.84, 121.92, 118.83, 118.29, 109.26, 55.71, 18.04 (Figure S18); HR-MS: [M+H]^+^ calculated 308.12812 *m*/*z*, found 308.12823 *m*/*z*.

#### 2-Hydroxy-*N*-(5-methoxy-2-methylphenyl)naphthalene-1-carboxamide (**17**)

Yield 76 %; mp 140-143 °C; IR (cm^–1^): 2945, 2833, 1642, 1585, 1513, 1450, 1437, 1350, 1291, 1278, 1146, 1030, 969, 896, 845, 816, 800, 768, 746, 719, 691, 679; ^1^H-NMR (DMSO-*d_6_*), *ẟ*: 10.17 (br.s, 1H), 9.71 (s, 1H), 7.83-7.89 (m, 3H), 7.50 (ddd, 1H, *J*=8.2 Hz, *J*=6.9 Hz, *J*=0.9 Hz), 7.31-7.36 (m, 1H), 7.26 (s, 1H), 7.25 (d, 1H, *J*=11.4 Hz), 7.16 (d, 1H, *J*=8.2 Hz), 6.74 (dd, 1H, *J*=8.2 Hz, *J*=2.7 Hz), 3.77 (s, 3H), 2.25 (s, 3H) (Figure S19); ^13^C-NMR (DMSO-*d_6_*), *ẟ*: 165.56, 157.35, 151.83, 137.20, 131.67, 130.75, 130.17, 127.95, 127.50, 126.90, 123.84, 123.69, 122.95, 118.35, 118.21, 111.14, 110.60, 55.17, 17.18 (Figure S20); HR-MS: [M+H]^+^ calculated 308.12812 *m*/*z*, found 308.12811 *m*/*z*.

#### *N*-(2-Chloro-5-methoxyphenyl)-2-hydroxynaphthalene-1-carboxamide (**18**)

Yield 87 %; mp 129-132 °C; IR (cm^–1^): 1635, 1583, 1512, 1456, 1435, 1413, 1353, 1273, 1231, 1213, 1190, 1168, 1150, 1126, 999, 968, 892, 860, 842, 812, 789, 743, 728, 677; ^1^H-NMR (DMSO-*d_6_*), *ẟ*: 10.48 (br.s, 1H), 9.87 (s, 1H), 8.04 (d, 1H, *J*=8.7 Hz), 7.89 (d, 1H, *J*=9.1 Hz), 7.85 (d, 1H, *J*=7.8 Hz), 7.70 (d, 1H, *J*=2.5 Hz), 7.50 (t, 1H, *J*=7.3 Hz), 7.44 (d, 1H, *J*=8.7 Hz), 7.34 (t, 1H, *J*=7.1 Hz), 7.25 (d, 1H, *J*=8.7 Hz), 6.84 (dd, 1H, *J*=8.9 Hz, *J*=2.5 Hz), 3.82 (s, 3H) (Figure S21); ^13^C-NMR (DMSO-*d_6_*), *ẟ*: 165.71, 158.22, 152.46, 135.82, 131.71, 131.08, 129.83, 128.03, 127.63, 127.05, 123.89, 123.09, 118.27, 117.29, 116.62, 111.55, 110.89, 55.56 (Figure S22); HR-MS: [M+H]^+^ calculated 328.073497 *m*/*z*, found 328.07379 *m*/*z*.

#### *N*-(5-Bromo-2-methoxyphenyl)-2-hydroxynaphthalene-1-carboxamide (**19**)

Yield 78 %; mp 231-234 °C; IR (cm^–1^): 1635, 1582, 1517, 1476, 1457, 1430, 1408, 1370, 1352, 1319, 1273, 1253, 1240, 1223, 1204, 1174, 1146, 1126, 1022, 966, 911, 879, 867, 816, 797, 752, 719, 700; ^1^H-NMR (DMSO-*d_6_*), *ẟ*: 10.51 (s, 1H), 9.57 (s, 1H), 8.54 (d, 1H, *J*=2.7 Hz), 8.00 (d, 1H, *J*=8.7 Hz), 7.88 (d, 1H, *J*=9.1 Hz), 7.84 (d, 1H, *J*=7.8 Hz), 7.48 (ddd, 1H, *J*=8.2 Hz, *J*=6.9 Hz, *J*=1.4 Hz), 7.32-7.36 (m, 1H), 7.30 (dd, 1H, *J*=8.7 Hz, *J*=2.7 Hz), 7.24 (d, 1H, *J*=9.1 Hz), 7.06 (d, 1H, *J*=8.7 Hz), 3.82 (s, 3H) (Figure S23); ^13^C-NMR (DMSO-*d_6_*), *ẟ*: 165.65, 152.41, 148.49, 131.71, 131.20, 129.22, 128.03, 127.67, 127.11, 126.49, 123.88, 123.12, 123.09, 118.25, 116.53, 113.16, 111.68, 56.15 (Figure S24); HR-MS: [M+H]^+^ calculated 372.022975 *m*/*z*, found 372.02365 *m*/*z*.

#### 2-Hydroxy*-N*-[2-methoxy-5-(trifluoromethyl)phenyl]naphthalene-1-carboxamide (**20**)

Yield 75 %; mp 205-208 °C; IR (cm^–1^): 3425, 3194, 1641, 1616, 1583, 1533, 1510, 1484, 1463, 1438, 1341, 1323, 1267, 1232, 1207, 1164, 1124, 1108, 1074, 1018, 969, 928, 895, 812, 792, 754, 711; ^1^H-NMR (DMSO-*d_6_*), *ẟ*: 10.53 (s, 1H), 9.72 (s, 1H), 8.73 (d, 1H, *J*=1.8 Hz), 8.01 (d, 1H, *J*=8.2 Hz), 7.89 (d, 1H, *J*=9.1 Hz), 7.85 (d, 1H, *J*=7.8 Hz), 7.46-7.53 (m, 2H), 7.34 (t, 1H, *J*=7.3 Hz), 7.27 (d, 1H, *J*=8.7 Hz), 7.25 (d, 1H, *J*=8.7 Hz), 3.91 (s, 3H) (Figure S25); ^13^C-NMR (DMSO-*d_6_*), *ẟ*: 165.94, 152.47, 151.88, 131.73, 131.24, 128.24, 128.06, 127.68, 127.15, 124.55 (q, *J*=271.7 Hz), 123.89, 123.15, 121.47 (q, *J*=4.8 Hz), 120.89 (q, *J*=31.8 Hz), 118.29, 117.22 (q, *J*=3.9 Hz), 116.55, 111.45, 56.28 (Figure S26); HR-MS: [M+H]^+^ calculated 362.099854 *m*/*z*, found 362.10028 *m*/*z*.

#### 2-Hydroxy*-N*-[4-methoxy-3-(trifluoromethyl)phenyl]naphthalene-1-carboxamide (**21**)

Yield 65 %; mp 178-181 °C; IR (cm^–1^): 3404, 3172, 1645, 1623, 1584, 1538, 1516, 1499, 1462, 1436, 1423, 1323, 1271, 1233, 1207, 1142, 1112, 1056, 1022, 972, 897, 815, 743, 661; ^1^H-NMR (DMSO-*d_6_*), *ẟ*: 11.52 (s, 1H), 10.15 (br.s, 1H), 8.22 (d, 1H, *J*=2.7 Hz), 7.96 (dd, 1H, *J*=8.9 Hz, *J*=2.5 Hz), 7.84-7.88 (m, 2H), 7.69 (d, 1H, *J*=8.7 Hz), 7.46 (ddd, 1H, *J*=8.3 Hz, *J*=7.0 Hz, *J*=1.1 Hz), 7.31-7.35 (m, 1H), 7.29 (d, 1H, *J*=9.1 Hz), 7.26 (d, 1H, *J*=9.1 Hz), 3.89 (s, 3H) (Figure S27); ^13^C-NMR (DMSO-*d_6_*), *ẟ*: 165.67, 152.79 (q, *J*=1.9 Hz), 151.71, 132.58, 131.37, 130.31, 127.99, 127.39, 127.06, 124.63, 123.65 (q, *J*=271.7 Hz), 123.40, 123.06, 118.34, 118.23, 117.61 (q, *J*=5.8 Hz), 116.61 (q, *J*=29.9 Hz), 113.41, 56.28 (Figure S28); HR-MS: [M+H]^+^ calculated 362.099854 *m*/*z*, found 362.10037 *m*/*z*.

#### 2-Hydroxy-*N*-[2-methyl-5-(trifluoromethyl)phenyl]naphthalene-1-carboxamide (**22**)

Yield 67 %; mp 143-146 °C; IR (cm^–1^): 3230, 2927, 1634, 1623, 1585, 1544, 1514, 1492, 1438, 1418, 1326, 1277, 1264, 1224, 1163, 1111, 1076, 972, 925, 883, 818, 761, 739, 710; ^1^H-NMR (DMSO-*d_6_*), *ẟ*: 10.26 (s, 1H), 10.03 (s, 1H), 8.06 (s, 1H), 7.85-7.89 (m, 3H), 7.48-7.53 (m, 3H), 7.33-7.37 (m, 1H), 7.26 (d, 1H, *J*=9.1 Hz), 2.42 (s, 3H) (Figure S29); ^13^C-NMR (DMSO-*d_6_*), *ẟ*: 165.96, 151.98, 137.12, 136.78, 131.57, 131.38, 130.45, 127.09, 127.48, 127.07, 126.76 (q, *J*=31.8 Hz), 124.29 (q, *J*=271.7 Hz), 123.58, 123.06, 121.54 (q, *J*=3.9 Hz), 121.46 (q, *J*=3.9 Hz), 118.33, 117.76, 18.03 (Figure S30); HR-MS: [M+H]^+^ calculated 346.10494 *m*/*z*, found 346.10507 *m*/*z*.

#### 2-Hydroxy-*N*-[4-methyl-3-(trifluoromethyl)phenyl]naphthalene-1-carboxamide (**23**)

Yield 60 %; mp 155-160 °C; IR (cm^–1^): 3053, 2674, 1634, 1598, 1584, 1539, 1513, 1502, 1436, 1419, 1328, 1273, 1241, 1207, 1167, 1140, 1123, 1107, 1053, 1042, 967, 809, 744, 682; ^1^H-NMR (DMSO-*d_6_*), *ẟ*: 10.62 (s, 1H), 10.17 (s, 1H), 8.28 (d, 1H, *J*=1.8 Hz), 7.84-7.90 (m, 3H), 7.68 (d, 1H, *J*=8.2 Hz), 7.44-7.48 (m, 1H), 7.42 (d, 1H, *J*=8.2 Hz), 7.31-7.35 (m, 1H), 7.26 (d, 1H, *J*=8.7 Hz), 2.42 (s, 3H) (Figure S31); ^13^C-NMR (DMSO-*d_6_*), *ẟ*: 166.05, 151.76, 137.85, 132.65, 131.32, 130.39, 130.28 (q, *J*=1.9 Hz), 128.00, 127.49 (q, *J*=28.9 Hz), 127.38, 127.09, 124.50 (q, *J*=273.6 Hz), 123.33, 123.07, 122.57, 118.33, 118.16, 116.15 (q, *J*=5.8 Hz), 18.23 (q, *J*=1.9 Hz) (Figure S32); HR-MS: [M+H]^+^ calculated 346.10494 *m*/*z*, found 346.10532 *m*/*z*.

#### 2-Hydroxy*-N*-[4-nitro-3-(trifluoromethyl)phenyl]naphthalene-1-carboxamide (**27**)

Yield 18 %; mp 157-160 °C; IR (cm^–1^): 3272, 1672, 1657, 1623, 1514, 1437, 1417, 1353, 1333, 1279, 1235, 1214, 1179, 1143, 1042, 968, 891, 834, 815, 804, 754, 744, 681; ^1^H-NMR (DMSO-*d_6_*), *ẟ*: 11.24 (s, 1H), 10.35 (br.s, 1H), 8.52 (s, 1H), 8.23-8.29 (m, 2H), 7.92 (d, 1H, *J*=9.1 Hz), 7.88 (d, 1H, *J*=8.2 Hz), 7.70 (d, 1H, *J*=8.7 Hz), 7.48 (ddd, 1H, *J*=8.6 Hz, *J*=7.0 Hz, *J*=1.4 Hz), 7.33-7.38 (m, 1H), 7.28 (d, 1H, *J*=9.1 Hz) (Figure S33); ^13^C-NMR (DMSO-*d_6_*), *ẟ*: 167.00, 152.14, 144.12, 141.45, 131.12, 131.03, 128.11, 127.85, 127.37, 127.35, 123.27, 123.13, 123.08 (q, *J*=32.8 Hz), 122.37, 122.11 (q, *J*=273.6 Hz), 118.27, 117.32 (q, *J*=6.1 Hz), 117.20 (Figure S34); HR-MS: [M+H]^+^ calculated 377.074368 *m*/*z*, found 377.07495 *m*/*z*.

### Lipophilicity determination by HPLC

An HPLC system Agilent 1200 equipped with a DAD detector (Agilent, Santa Clara, CA, USA) was used. A chromatographic column Symmetry^®^ C_18_ 5 μm, 4.6×250 mm, part No. WAT054275 (Waters Corp., Milford, MA, USA) was used. The HPLC separation process was monitored and evaluated with EZChrom Elite software ver. 3.3.2 (Agilent) [56]. Isocratic elution by a mixture of MeOH *p.a.* (72 %) and H_2_O-HPLC Mili-Q grade (28 %) as a mobile phase was used for the determination of capacity factor *k*. Isocratic elution by a mixture of MeOH *p.a.* (72 %) and acetate-buffered saline (pH 7.4 and pH 6.5) (28 %) as a mobile phase was used for the determination of distribution coefficients expressed as *D*_7.4_ and *D*_6.5_. The total flow of the column was 1.0 mL min^-1^, the injection volume was 20 μL, the column temperature was 40 °C, and the sample temperature was 10 °C. The detection wavelength of 210 nm was chosen. A KI methanolic solution was used to determine the dead times (*t*_D_). Retention times (*t*_R_) were measured in minutes. The capacity factors *k* were calculated according to the formula *k* = (*t*_R_–*t*_D_)/*t*_D_, where *t_R_* is the retention time of the solute, and *t_D_* is the dead time obtained using an unretained analyte. The distribution coefficients *D*_pH_ were calculated according to the formula *D*_pH_ = (*t*_R_–*t*_D_)/*t*_D_. Each experiment was repeated three times. The experimental values of lipophilicity of individual compounds are shown in Table 1.

### Antibacterial screening

*In vitro* antibacterial activity of the synthesized compounds was evaluated against representatives of multidrug-resistant bacteria, three clinical isolates of methicillin-resistant *S. aureus*: clinical isolate of animal origin, MRSA 63718 [57] (Department of Infectious Diseases and Microbiology, Faculty of Veterinary Medicine, University of Veterinary Sciences Brno, Czech Republic), and MRSA SA 630 and MRSA SA 3202 [57] (National Institute of Public Health, Prague, Czech Republic), both of human origin. These three clinical isolates, carrying the *mecA* gene [58], were classified as vancomycin-susceptible (but with higher MIC of vancomycin equal to 2 μg mL^-1^ (VA2-MRSA) within the susceptible range for MRSA 63718) methicillin-resistant *S. aureus* (VS-MRSA) [57]. Vancomycin- and methicillin-susceptible *S. aureus* ATCC 29213 and vancomycin-susceptible *Enterococcus faecalis* ATCC 29212, obtained from the American Type Culture Collection, were used as the reference and quality control strains. Three *vanA* gene-carrying vancomycin-resistant isolates of *E. faecalis* (VRE 342B, VRE 368, VRE 725B) were provided by Oravcova *et al.* [59]. In addition, all the prepared compounds were tested against the Gram-negative bacteria *E. coli* ATCC 25922 (American Type Culture Collection).

The minimum inhibitory concentrations (MICs) were evaluated using the microtitration broth method according to the CLSI [60,61], with some modifications. The compounds were dissolved in DMSO (Sigma, St. Louis, MO, USA) to get a concentration 10 μg mL^-1^ and diluted in a microtitration plate in an appropriate medium, *i.e.* Cation Adjusted Mueller–Hinton Broth (CaMH, Oxoid, Basingstoke, UK) for staphylococci, *E. coli*; and Brain Heart Infusion Broth (BHI, Oxoid) for enterococci to reach the final concentration of 256 to 0.125 μg mL^-1^. Microtitre plates were inoculated with test microorganisms so that the final concentration of was 10^5^ bacterial cells in a microtiter plate. Ampicillin and ciprofloxacin (Sigma) were used as reference drugs. A drug-free control and a sterility control were included. The plates were incubated for 24 h at 37 °C for all the tested bacteria. After static incubation in the darkness in an aerobic atmosphere, the MIC was visually evaluated as the lowest concentration of the tested compound, which completely inhibited the growth of the microorganism. The experiments were repeated three times. The results are summarized in Table 2.

**Table 2.** *In vitro* activities (MICs) of investigated compounds against bacteria compared to ampicillin (AMP) and ciprofloxacin (CPX)

Comp.	R	MIC, μM								
		*S. aureus*	MRSA 63718	MRSA SA 630	MRSA SA 3202	*E. faecalis*	VRE 342B	VRE 725B	VRE 368	*E. coli*
**1**	H	>972	>972	243	122	>972	>972	>972	>972	972
**2**	2-OCH_3_	>873	>873	>873	>873	>873	>873	>873	>873	873
**3**	3-OCH_3_	436	436	873	218	>873	>873	>873	>873	873
**4**	4-OCH_3_	>873	>873	>873	>873	>873	>873	>873	>873	873
**5**	2,5-OCH_3_	792	792	792	792	792	792	792	792	792
**6**	3,5-OCH_3_	198	396	396	198	792	792	792	792	792
**7**	3,4,5-OCH_3_	838	838	838	419	724	724	724	724	838
**8**	2-CH_3_	462	462	462	462	462	462	462	462	462
**9**	3-CH_3_	231	462	231	231	462	462	462	462	462
**10**	4-CH_3_	231	462	462	462	462	462	462	462	462
**11**	2,5-CH_3_	110	110	110	110	879	879	879	879	879
**12**	2,6-CH_3_	439	879	439	439	879	879	879	879	879
**13**	3,5-CH_3_	54.9	54.9	54.9	54.9	879	879	879	879	879
**14**	2,4,6-CH_3_	838	838	838	419	838	838	838	838	838
**15**	2-OCH_3_-5-CH_3_	833	833	833	833	833	833	833	833	833
**16**	2-OCH_3_-6-CH_3_	833	833	833	833	416	208	833	208	833
**17**	2-CH_3_-5-OCH_3_	104	416	208	208	833	833	833	833	833
**18**	2-Cl-5-OCH_3_	48.8	195	48.8	97.6	781	781	781	781	781
**19**	2-OCH_3_-5-Br	688	688	688	688	688	688	688	688	688
**20**	2-OCH_3_-5-CF_3_	709	709	709	709	709	709	709	709	709
**21**	3-CF_3_-4-OCH_3_	88.6	177	88.6	88.6	88.6	177	88.6	88.6	709
**22**	2-CH_3_-5-CF_3_	2.90	5.79	2.90	2.90	46.3	92.6	92.6	46.3	23.2
**23**	3-CF_3_-4-CH_3_	23.2	371	92.7	46.3	741	741	741	741	741
**24**	2-NO_2_	26.0	415	104	51.9	830	830	830	830	830
**25**	3-NO_2_	208	26.0	208	208	830	830	830	830	830
**26**	4-NO_2_	>830	830	415	104	415	830	830	830	830
**27**	3-CF_3_-4-NO_2_	0.332	1.33	1.33	1.33	21.3	42.5	42.5	21.3	680
**AMP**	–	5.72	45.8	45.8	45.8	2.81	11.5	11.5	11.5	45.8
**CPX**	–	0.75	24.2	24.2	24.2	1.51	3.02	193	3.02	0.377

### Antimycobacterial screening

The evaluation of *in vitro* antimycobacterial activity of the compounds was performed against *Mycobacterium tuberculosis* ATCC 25177/H_37_Ra, *Mycobacterium kansasii* DSM 44162 and *Mycobacterium smegmatis* ATCC 700084.

The broth dilution micro-method in Middlebrook 7H9 medium (Difco, Lawrence, KS, USA) supplemented with ADC Enrichment (Difco) was used to determine the minimum inhibitory concentration (MIC) as previously described [61]. The compounds were dissolved in DMSO (Sigma), and the final concentration of DMSO did not exceed 2.5 % of the total solution composition. The final concentrations of the evaluated compounds, ranging from 256 to 0.125 μg mL^-1^, were obtained by twofold serial dilution of the stock solution in a microtiter plate with a sterile medium. Bacterial inocula were prepared by transferring colonies from culture to sterile water. The plate was inoculated by tested microorganisms. The final concentration of bacterial cells was 1.5×10^6^ for *M. tuberculosis* and 10^5^ cells in a microtiter plate for other mycobacteria. Isoniazid and rifampicin (Sigma) were used as reference antimycobacterial drugs. Drug-free controls, sterility controls, and controls consisting of medium and DMSO alone were included. The plates were incubated for a defined time at an appropriate temperature (3 days at 37 °C for *M. smegmatis*, and 14 days at 37 °C for *M. tuberculosis* and *M. kansasii*). After incubation, the MIC was visually evaluated as the lowest concentration of the tested compound, which completely inhibited the growth of the microorganism. The MICs against *M. tuberculosis* were evaluated by Alamar blue (Oxoid). After incubation, 10 % of Alamar blue was added to each well, and the plate was incubated for 24 h. The MIC values were assessed as the lowest concentration of the tested compounds, which prevented changing blue resazurin to pink resorufin. The experiments were repeated three times. The minimum inhibitory concentrations (MICs) were defined as the lowest concentration of the compound at which no visible bacterial growth was observed. The MIC value is routinely and widely used in bacterial assays and is a standard detection limit according to the CLSI [60]. The results are summarized in Table 3.

**Table 3.** *In vitro* activities (MICs) of investigated compounds against mycobacteria compared to isoniazid (INH), rifampicin (RIF), and *in vitro* cytotoxicity assay (LC_50_) of choice compounds on human monocytic leukemia THP-1 cells compared to oxaliplatin (OXP), camptothecin (CMP)

Comp.	R	MIC, μM			LC_50_, μM
		*M. tuberculosis*	*M. kansasii*	*M. smegmatis*	
**1**	H	486	15.2	486	>20 [43]
**2**	2-OCH_3_	>873	>873	>873	–
**3**	3-OCH_3_	218	109	218	–
**4**	4-OCH_3_	109	218	436	–
**5**	2,5-OCH_3_	396	792	792	–
**6**	3,5-OCH_3_	396	98.9	396	>30
**7**	3,4,5-OCH_3_	210	838	210	–
**8**	2-CH_3_	231	115	462	–
**9**	3-CH_3_	231	115	231	–
**10**	4-CH_3_	231	115	>923	–
**11**	2,5-CH_3_	439	110	220	–
**12**	2,6-CH_3_	439	879	879	–
**13**	3,5-CH_3_	439	110	220	>30
**14**	2,4,6-CH_3_	210	838	210	–
**15**	2-OCH_3_-5-CH_3_	416	833	833	–
**16**	2-OCH_3_-6-CH_3_	416	833	416	–
**17**	2-CH_3_-5-OCH_3_	416	833	416	–
**18**	2-Cl-5-OCH_3_	391	391	781	–
**19**	2-OCH_3_-5-Br	344	688	688	–
**20**	2-OCH_3_-5-CF_3_	354	709	709	–
**21**	3-CF_3_-4-OCH_3_	354	709	177	–
**22**	2-CH_3_-5-CF_3_	46.3	46.3	23.2	>30
**23**	3-CF_3_-4-CH_3_	371	741	46.3	–
**24**	2-NO_2_	104	51.9	208	>20 [43]
**25**	3-NO_2_	104	104	208	>20 [43]
**26**	4-NO_2_	104	415	208	2.5±0.1 [43]
**27**	3-CF_3_-4-NO_2_	85.0	21.3	21.3	>30
**INH**	–	36.5	233	117	–
**RIF**	–	9.71	0.150	19.4	–
**OXP**	–	–	–	–	1.7±0.6
**CMP**	–	–	–	–	0.16±0.07

### Cytotoxicity assay

Cytotoxicity of the compounds was determined using an LDH assay kit (Roche Diagnostics, Mannheim, Germany) as described previously [43,54]. Human monocytic leukemia THP-1 cells (European Collection of Cell Cultures, Salisbury, UK) were exposed for 24 h at 37 °C to various compound concentrations ranging from 0.37 to 30 μM in RPMI 1640 medium. For LDH assays, cells were seeded into 96-well plates (5×10^4^ cells/well in 100 μL culture medium) in triplicate in serum-free RPMI 1640 medium and measurements were taken 24 h after the treatment with the compounds. The maximum concentration of DMSO (Sigma) in the assays never exceeded 0.1 %. Oxaliplatin and camptothecin (Sigma) were used as reference drugs. The median lethal dose values, LC_50_, were deduced through the production of a dose-response curve. All data from three independent experiments were evaluated using GraphPad Prism 5.00 software (GraphPadSoftware, San Diego, CA, USA) [62]. The results are summarized in Table 3.

## Results and discussion

### Chemistry

**Scheme 1.**
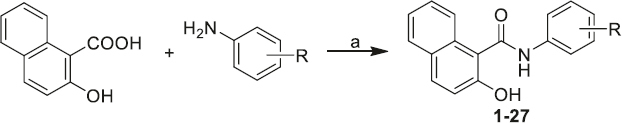
Synthesis of ring-substituted 2-hydroxynaphthalene-1-carboxanilides **1**-**27**. *Reagents and conditions*: (a) PCl_3_, chlorobenzene, microwave synthesis (500 W, 130 °C, 15 min)

The compounds were prepared by a simple (click chemistry method) but innovative microwave synthesis from commercially available building blocks (2-hydroxy-1-naphthoic acid and multisubstituted anilines) in anhydrous chlorobenzene in the presence of PCl_3_. The synthesis is depicted in Scheme 1 and a list of all studied compounds is given in Table 1. The unsubstituted derivative **1** and monosubstituted compounds **2**-**4**, **8**-**10**, **24**-**26** have already been described by Gonec *et al.* [43] but are listed here for the completeness of the entire study.

Since the basis for understanding the behavior of bioactive molecules is the knowledge of their lipo-hydrophilic properties, lipophilicity parameters were determined for all compounds, namely log *k* (logarithm of the capacity factor) and log *D* (logarithm of the distribution coefficient) at physiological pH 6.5 and 7.4.

The capacity factor and distribution coefficients were measured by RP-HPLC on a C18 column with methanol as an organic modifier of the mobile phase. In addition to the experimental lipophilicities, predicted log *P* values were also calculated using ACD/Percepta [55], see Table 1. In addition to lipophilicity, Table 1 also shows the predicted (ACD/Percepta [55]) electronic *σ*_(Ar)_ parameters of the whole substituted anilide ring, characterizing the electron-withdrawing or donating ability of the molecular system. The values of *σ*_(Ar)_ are found in a wide range from 0.01 to 1.36, so the compounds contain substituents with both electron-donating and slightly electron-withdrawing properties.

**Figure 1.**
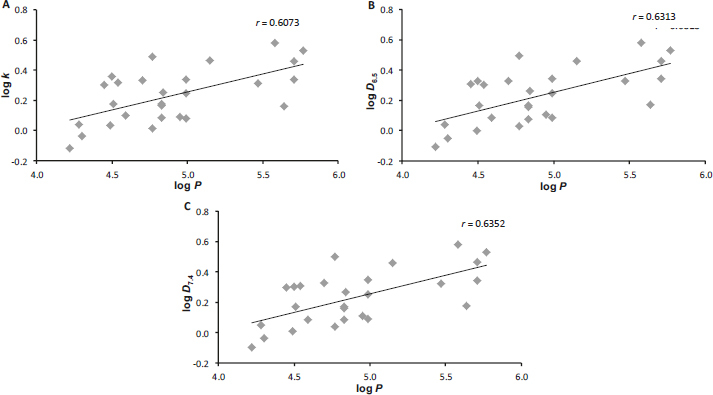
Comparison of experimentally determined values of log *k* (A), log *D*_6.5_ (B), and log *D*_7.4_ (C) with calculated log *P* (ACD/Percepta [55]) of carboxanilides **1**-**27**

**Figure 2.**
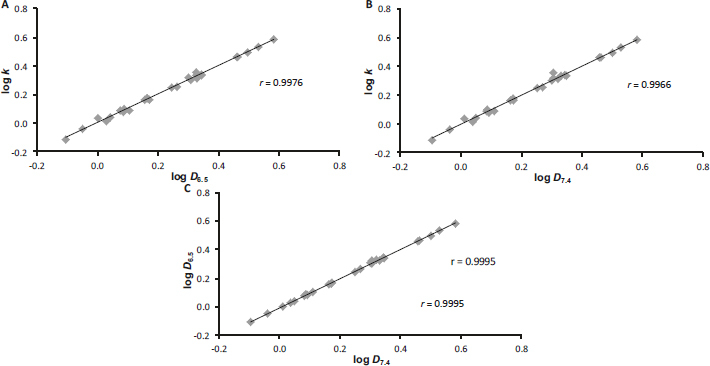
Cross-correlations of experimentally determined values of log *k* versus log *D*_6.5_ (A), log *k* versus log *D*_7.4_ (B) and log *D*_6.5_ versus log *D*_7.4_ (C) of carboxanilides 1-27

The graphs in Figure 1 show the correlations between the experimental and calculated lipophilicity values, and as can be seen from the correlation coefficients *r*, which range from 0.60 to 0.63 (*n* = 27), the agreement is small, which probably indicates a significant influence of the free phenol group, which the software cannot capture. On the other hand, the graphs in Figure 2 illustrate the relationships between log *k* and log *D*, which, according to the correlation coefficient *r* of ca. 0.99 (*n* = 27), are very good.

**Figure 3.**
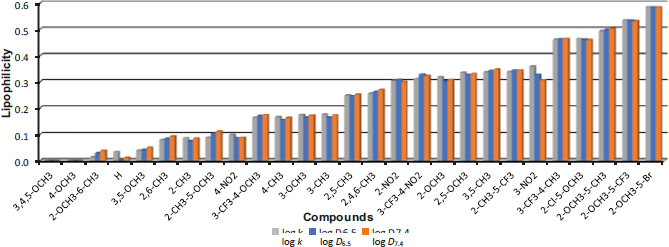
Order of individual derivatives arranged according to increasing log k values

Figure 3 shows the order of the compounds according to the increasing log *k* value. The least lipophilic are methoxylated derivatives **7** (R = 3,4,5-OCH_3_) and **4** (R = 4-OCH_3_), while the most lipophilic are compounds **20** (R = 2-OCH_3_-5-CF_3_) and **19** (R = 2-OCH_3_-5-Br). The unsubstituted derivative **1** (the fourth least lipophilic compound in this series) showed the largest deviation between the log *k* and log *D* values.

Regarding all these observations, it should be summarized that for these 2-hydroxynaphthalene-1-carboxamides on the ring-multisubstituted anilide, standard commercially available lipophilicity prediction programs are unable to provide relevant data due to the high incidence of intra- and intermolecular interactions. On the other hand, the presence of an ionizable acidic phenolic group in the vicinity of the amide bond does not cause significant differences in the experimental values obtained for different mobile phase properties/compositions.

### In vitro biological activities

The biological properties were assessed for *in vitro* antibacterial and antimycobacterial activity. In addition, all the compounds were also evaluated for their cytotoxicity against human monocytic leukemia THP-1 cells. The selection of the studied bacterial strains was adopted following the CLSI (National Committee for Clinical Laboratory Standards) international reference methodologies [63], *i.e*. standardization. For this purpose, universally sensitive collection strains from ATCC (*Staphylococcus aureus* ATCC 29213, *Enterococcus faecalis* ATCC 29212 and *Escherichia coli* ATCC 25922) were selected. The second aspect of strain selection was the current state of occurrence of strains with an epidemiologically significant type of resistance, represented by clinical isolates of human and veterinary origin, *i.e*. different sequence types limited to human and animal populations, *e.g.* methicillin-resistant *Staphylococcus aureus* (MRSA) SA 3202, SA 630, 63718 isolates carrying the *mecA* gene [57]. In the case of vancomycin-resistant *E. faecalis* (VRE) 342B, 368, and 725B isolates carrying the *vanA* gene [59], these were isolates from wild birds colonized from US hospital wastewater, as confirmed. Therefore, it can be concluded that the tested strains differed in the spectrum of antibiotic resistance, genetic makeup, and probably accessory genome. Activities are expressed as minimum inhibitory concentrations (MICs), as shown in Table 2.

To obtain a comprehensive overview of the antibacterial properties of the investigated compounds, all derivatives were tested *in vitro* against *Mycobacterium tuberculosis* ATCC 25177/H_37_Ra, *Mycobacterium kansasii* DSM 44162 and *Mycobacterium smegmatis* ATCC 700084; activities are expressed as MICs as reported in Table 3. In order to reduce risks, a replacement of model pathogens is commonly used in basic laboratory screening. For *M. tuberculosis*, avirulent strain H_37_Ra is used, which has a similar pathology as *M. tuberculosis* strains infecting humans and, thus, represents a good model for testing antitubercular agents [64]. The genus *Mycobacterium* is a closely related group of fast- and slow-growing species. In addition to *M. tuberculosis*, there are a number of other so-called atypical (non-tuberculous) mycobacteria, important environmental pathogens, that cause a wide range of diseases (pulmonary diseases, lymphadenitis, skin and soft tissue diseases, gastrointestinal and skeletal infections), especially in immunocompromised patients [65-68]. These non-tuberculous strains include the fast-growing, e.g. *M. smegmatis* [69,70] and the slow-growing, *e.g. M. kansasii* [71,72].

#### Antimicrobial activities

Looking at Tables 2 and 3, it should be noted that the compounds had very limited activity. Of all the studied derivatives, only a total of 7 compounds (**13**, **18**, **21**-**24** and **27**) showed some activity, with **13** (R = 3,5-CH_3_), **22** (R = 2-CH_3_-5-CF_3_) and **27** (R = 3-CF_3_-4-NO_2_) being truly effective. Of this number, it is, of course, not possible to meaningfully discuss structure-activity relationships. On the other hand, it is important to note that despite the small number of active compounds, their efficacy, when they were active, was at the level of clinically used drugs.

Compounds **13**, **22** and **27** were active against *S. aureus*/MRSA, two of which (**22**, **27**) were also active against *E. faecalis*/VRE. Since the compounds were active against both the collection strains and resistant isolates, it is possible to speculate on a specific mechanism of action different from that of beta-lactam or quinolone antibiotics and the demonstrated resistance. The lower potency against *E. faecalis*/VRE compared to *S. aureus*/MRSA is likely due to the overall higher resistance of *E. faecalis*/VRE, including their ability to be facultative anaerobic bacteria [73-76]. It should be added that compound **22** was the only one that surprisingly showed activity against the Gram-negative collection strain *E. coli*. Several compounds also demonstrated activity against mycobacteria. Derivatives **22** and **27** were active against all three evaluated mycobacterial species. In addition, **23** (R = 3-CF_3_-4-CH_3_) showed activity against the fast-growing *M. smegmatis* and **24** (4-NO_2_) also against the slow-growing *M. kansasii*.

Considering the activities of previously published monosubstituted derivatives, 2-hydroxy-*N*-(2-nitrophenyl)naphthalene-1-carboxamide (**24**) was the most antimicrobially active (data see Tables 2 and 3), followed by *N*-(4-bromophenyl)-2-hydroxynaphthalene-1-carboxamide and 2-hydroxy-*N*-(4-trifluoromethylphenyl)naphthalene-1-carboxamide against MRSA SA 630 and SA 3202 (MICs 47 and 94 μM, respectively) and *M. kansasii* (MICs 93 and 23 μM, respectively) [43]. Therefore, it can be stated that overall the previously described compounds had even more limited effects than these disubstituted derivatives.

Suppose these new observations are generalized from the point of view of the significance of substituents in the anilide part of the molecule. In that case, it is necessary to state that substitution with methoxy groups is completely disadvantageous for any antimicrobial activity. The situation changes slightly if the methoxy moiety is replaced by a methyl group; compare **5** (R = 2,5-OCH_3_) and **6** (R = 3,5-OCH_3_) with **11** (R = 2,5-CH_3_) and **13** (R = 3,5-CH_3_). Similar findings were published recently [52,54]. Disubstitution with 3,5-CH_3_ led to an increase in antistaphylococcal activity. The subsequent combination of methyl with a CF_3_ group in the *meta* position (compounds **23** and especially **22**) resulted in a further significant increase and, above all, the extension of activity to *E. faecalis*/VRE, Gram-negative bacteria and mycobacteria (compare **20** (R = 2-OCH_3_-5-CF_3_ and **22** (R = 2-CH_3_-5-CF_3_)). The positive influence of the CF_3_ moiety on the potency and extension of antimicrobial activity was also observed in the nitrated disubstituted derivative **27** (compared with compound **26**). These observations (the advantage of combining CH_3_ or NO_2_ with CF_3_) are completely new and have not been found in previously studied isomers [37,54].

**Figure 4.**
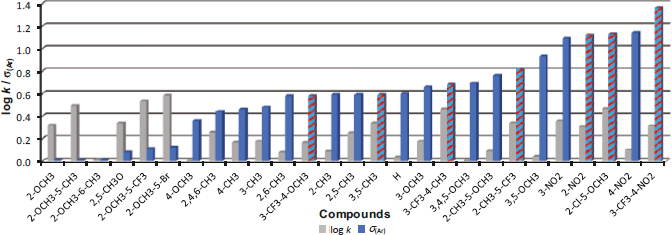
Order of individual derivatives arranged according to increasing electron *σ*_(Ar)_ parameter compared to log *k* values. Compounds with some effect are hatched in blue-red

The individual derivatives ordered by increasing electron σ_(Ar)_ parameter are shown in Figure 4, where log *k* values are also given for comparison. The hatched bars in the graph indicate seven compounds (*i.e*. **13**, **18**, **21**-**24** and **27**) demonstrating some activity. The first more significant individual effect was achieved at a log *k* value of 0.16 (compound **21**, R = 3-CF_3_-4-OCH_3_). On the other hand, the activity disappeared at log *k* values of 0.46 (compounds **23** (R = 3-CF_3_-4-CH_3_), **18** (R = 2-Cl-5-OCH_3_)). The highest/widest activity was achieved with a log *k* value higher than 0.31 (**27**, R = 3-CF_3_-4-NO_2_) and lower than 0.34 (**22**, R = 2-CH_3_-5-CF_3_). So, it is evident that lipophilicity plays a secondary role.

On the other hand, the potency and wide antimicrobial activity are much more influenced by the electron σ_(Ar)_ parameter; it is advantageous if it is higher than 0.58 (derivative **21** (R = 3-CF_3_-4-OCH_3_), see Figure 4). In accordance with the Michael acceptor theory, the higher the substituent causes an electron deficit, the better. Therefore, it seems that this is a substituent-dependent activity (dependent on the type and position of the substituents), where the electron-deficient state of the molecule (characterized by the magnitude of the parameter σ_(Ar)_) plays a significant role, similarly as described, *e.g.* in [29,43,77-80]. Thus, it can be said that the investigated effective compounds meet the definition of so-called Michael acceptors, where in addition to suitable lipophilicity for penetration through the bacterial wall, the overall electron deficit in the molecule is key for efficacy and binding to targets (bacterial biomolecules) appears to occur primarily through polar groups and a planar π-π system.

#### Cytotoxicity

Preliminary *in vitro* cytotoxicity screening of selected active compounds was performed using the THP-1 cell line. Cytotoxicity was expressed as LC_50_ value (lethal concentration for 50 % of the cell population), see Table 3. Treatment with 30 μM of the new compounds did not result in a significant lethal effect on THP-1 cells (*e.g.* LC_50_ values of oxaliplatin and camptothecin were 1.7±0.64 and 0.16±0.07 μM). Among nine previously synthesized monosubstituted 2-hydroxynaphthalene-1-carboxanilides, the cytotoxicity of four of them was previously examined. A significant lethal effect was detected only in the case of compound **26** (LC_50_ = 2.5 μM), while compounds **1**, **24**, and **25** did not show significant cytotoxicity (LC_50_ > 20 μM) [43]. Based on these observations, it can be concluded that tested substances except **26** can be considered non-toxic substances for the subsequent design of new antimicrobial agents.

### Computational ADME Properties

In the early stage of the drug discovery process, especially orally administered drugs, it is extremely important to perform at least indicative ADME profiling to provide critical information about the basic behavior of a potential drug in the body. Basic information obtained from such studies helps guide further structural optimizations to obtain molecules with more favorable pharmacokinetic parameters while maintaining the existing efficiency potential. A variety of software is advantageously used for basic ADME screening, and ADME profiling is subsequently verified for selected drug candidates in preclinical and clinical studies [81-83]. The Lipinski Rule of Five (Ro5) is one of the most accepted recommendations concerning the physicochemical parameters of biologically active compounds, and all medicinal chemists try to follow it when designing molecules [84]. Ro5 contains the limits of specific molecular descriptors (MW <500, log *P* <5, HBD <5, HBA <10) set based on experimentally and statistically obtained results so that a compound that meets this recommendation has a higher chance of becoming a drug. However, a good drug-like score does not make a molecule a drug, and vice versa [85,86]. In addition, information has been added about compounds related to the Veber rule, which states that a compound with 10 or fewer rotational bonds (RB) and a polar surface area (TPSA) of no higher than 1.4 nm^2^ (140 Å^2^) should have good oral bioavailability [81,87]. It is clear that ADMET-friendly properties, such as lipophilicity, polar surface area, *etc*, are important in the context of specific ligand-receptor interactions.

The following Table 4 shows the predicted ADME-influencing properties of the most effective compounds (**13**, **22**, **27**). The compounds are rather rigid and expected to be nearly planar [54]. All contain a free acidic phenolic group, which is crucial for biological activity [40,41,43], meaning that these acidic compounds in plasma bind predominantly to human serum albumin [88]. In addition to Ro5 parameters, other parameters, such as intestinal absorption and permeation into the brain (the effectiveness of antibiotics in the brain is important), are listed in Table 4. All the parameters were predicted using the commercially available program ACD/Percepta [55].

**Table 4.** Values of parameters characterizing physicochemical properties of discussed effective 2-hydroxynaphthalene-1-carboxanilides predicted using ACD/Percepta [55]

Comp.	MW	log *P*	HBD	HBA	RB	TPSA, nm^2^	Parachor, cm^3^	*k*_a_ / min^-1^	log BB	log PS	Brain plasma equilibrim rate
**13**	291.34	4.99	2	3	2	0.49	641.03	0.055	0.54	-1.1	-3.2 (brain penetration sufficient for CNS activity)
**22**	345.32	5.71	2	3	3	0.49	660.59	0.055	-0.11	-1.2	-3.5 (probably CNS inactive due to low brain penetration)
**27**	376.29	5.47	2	6	4	0.98	678.43	0.055	-0.24	1.3	-3.5 (probably CNS inactive due to low brain penetration)

All the discussed agents have molecular weights (MW) significantly <500. On the other hand, the compounds have rather higher lipophilicity (log *P* ≥5). All the compounds meet the criteria for the number of H-bond donors (HBD) and acceptors (HBA). The number of rotatable bonds (RB) is in the narrow range of 2 to 4. The topological polar surface area (TPSA) has been recognized as a good indicator of intestinal drug absorption (TPSA <1.2-1.4 nm^2^ (<120-140 Å^2^)) and blood-brain barrier (BBB) penetration (TPSA <0.6 nm^2^ (<60 Å^2^)) [87,89,90]. The TPSA value of 0.49 nm^2^ (49 Å^2^) indicates that compounds **13** and **22** should have good intestinal absorption as well as adequate BBB penetration. On the other hand, the TPSA = 0. 98 nm^2^ (98 Å^2^) for compound **27** suggests only good intestinal absorption. The predicted value of the absorption rate in the jejunum (*k*_a_ = 0.055 min^-1^) is the same for all derivatives. A remarkable prediction was made by ACD/Percepta [55] for blood-brain barrier (BBB) permeation. In general, log BB ≥ 0.3 is for BBB permeable drugs and log BB ≤ -0.3 is for impermeable drugs [91]. The log BB values of -0.11 (**22**) and -0.24 (**27**), respectively, indicate that both compounds are probably CNS inactive due to low brain penetration (as already suggested by the TPSA value for **27**). Conversely, the log BB = 0.54 for **13** indicates good BBB penetration. More informative are the values of the permeability surface-area product (expressed as log PS) [92] in the range of -1.2 to 1.3. The brain plasma equilibrium rate predicted by ACD/Percepta [55] for compound **13** is -3.2, suggesting that this compound may achieve sufficient brain penetration for CNS activity.

In summary, after preliminary *in silico* ADME screening using commercially available software, it can be assumed that investigated compounds **13**, **22**, and **27** should have suitable physicochemical parameters for adequate bioavailability in the body. Unfortunately, for the most effective of the compounds **37**, probably due to the presence of the NO_2_ group, BBB penetration was not predicted. Of course, much more accurate results could be obtained using other experiments [93].

## Conclusions

A series of nine previously synthesized monosubstituted 2-hydroxynaphthalene-1-carboxanilides was enriched with seventeen new di- and trisubstituted 2-hydroxynaphthalene-1-carboxanilides and all the compounds were tested for their *in vitro* antibacterial and antimycobacterial activity. Only five compounds showed antimicrobial activity, and three of them were comparable to drugs that were clinically used. *N*-(3,5-Dimethylphenyl)-2-hydroxynaphthalene-1-carboxamide (**13**) was active only against *S. aureus* and MRSA isolates, 2-hydroxy-*N*-[2-methyl-5-(trifluoromethyl)phenyl]naphthalene-1-carboxamide (**22**) and 2-hydroxy*-N*-[4-nitro-3-(trifluoromethyl)phenyl]naphthalene-1-carboxamide (**27**) were active across the entire spectrum of tested bacteria/mycobacteria, both against susceptible and resistant isolates. Compound **22** was even active against Gram-negative *E. coli*. These active agents showed no *in vitro* cytotoxicity against THP-1 cells up to a concentration of 30 μM. Based on preliminary *in silico* ADME screening using commercially available software, it can be assumed that investigated compounds **13**, **22** and **27** should have suitable physicochemical parameters for adequate bioavailability in the body. From the observations, it can be stated that the two most active compounds **22** and **27** are substituted with a CF_3_ moiety in the *meta* position of the anilide, in addition to the methyl or nitro group. The CF_3_ moiety thus proved to be a necessary prerequisite for antimicrobial activity in structures of this type. It was indirectly verified that the biological effects of this scaffold are based on the Michael acceptor theory. The CF_3_ moiety, primarily due to its electron-withdrawing properties, meets the definition of Michael acceptors, where the overall electron deficit in the molecule (caused by appropriate substitution) appears to be essential for the expected binding to targets in the bacterial cell, resulting in antimicrobial activity. Given the overall structure of the investigated compounds, multiple mechanisms of action can be assumed; efforts to discover them will subsequently be carried out using proteomic and molecular biological experiments.

## Supplementary material

^1^H and ^13^C NMR spectra of the new discussed compounds are available from the corresponding author on request, or at https://pub.iapchem.org/ojs/index.php/admet/article/view/2642.


