# Supplementary material for: Antimicrobial and ADME properties of methoxylated, methylated and nitrated 2-hydroxynaphthalene-1 carboxanilides
Source: ADMET DMPK. 2025 Feb 8;13(1):2642. doi: 10.5599/admet.2642 (PMC11954145; doi:10.5599/admet.2642)
Supplement: Supplementary file 1 [file ADMET-13-2642-S1.pdf]

ADMET & DMPK 13(1) (2025) S2642

Open Access : ISSN : 1848-7718

http://www.pub.iapchem.org/ojs/index.php/admet/index

Supplementary material to

Antimicrobial and ADME properties of methoxylated, methylated and nitrated 2-hydroxynaphthalene-1 carboxanilides

Lucia Vrablova[1], Tomas Gonec[2], Tereza Kauerova[3], Michal Oravec[4], Izabela Jendrzejewska[5], Peter Kollar[3], Alois Cizek[6] and Josef Jampilek[1,7]

[1]*Department of Analytical Chemistry, Faculty of Natural Sciences, Comenius University, Ilkovicova 6, 84215 Bratislava, Slovakia*

[2]*Department of Chemical Drugs, Faculty of Pharmacy, Masaryk University, Palackeho tr. 1946/1, 61200 Brno, Czech Republic*

[3]*Department of Pharmacology and Toxicology, Faculty of Pharmacy, Masaryk University, Palackeho tr. 1946/1, 61200 Brno, Czech Republic*

[4]*Global Change Research Institute CAS, Belidla 986/4a, 60300 Brno, Czech Republic*

[5]*Institute of Chemistry, University of Silesia, Bankowa 12, 40007 Katowice, Poland*

[6]*Department of Infectious Diseases and Microbiology, Faculty of Veterinary Medicine, University of Veterinary Sciences Brno, Palackeho tr. 1946/1, 61242 Brno, Czech Republic*

[7]*Department of Chemical Biology, Faculty of Science, Palacky University Olomouc, Slechtitelu 27, 77900 Olomouc, Czech Republic*

ADMET & DMPK **13(1)** (2025) 2642; https://doi.org/10.5599/admet.2642

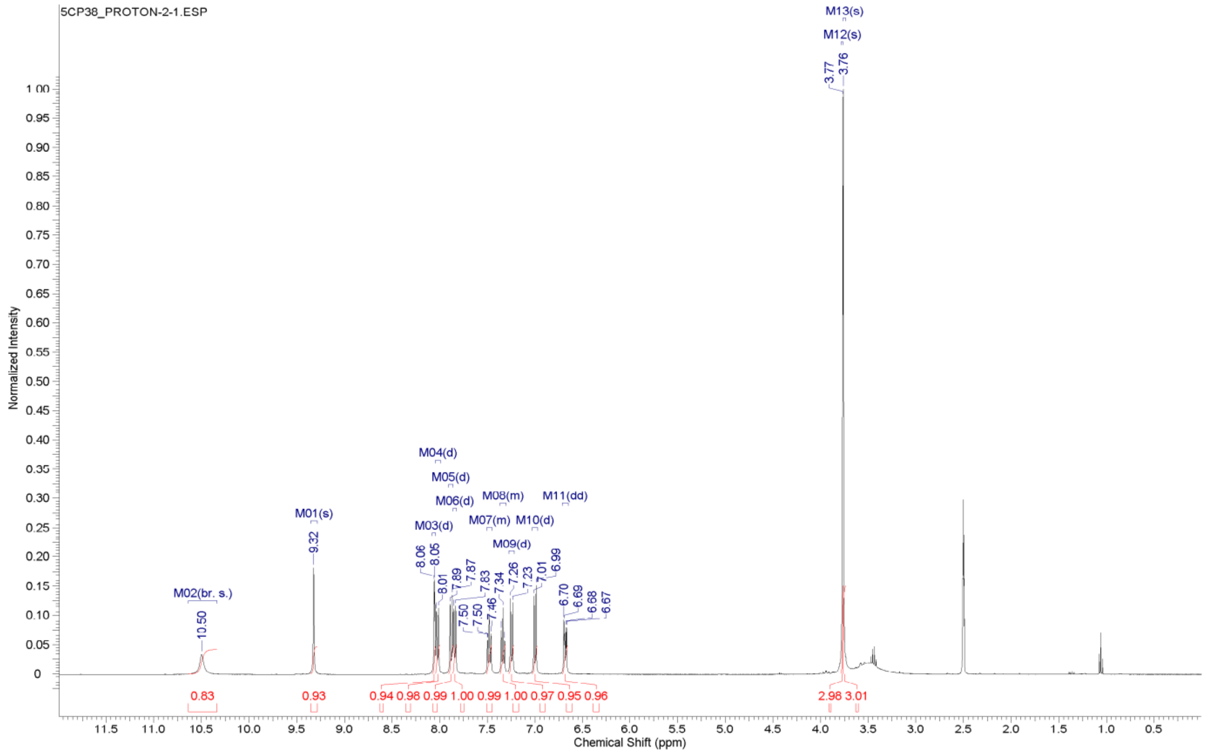

Figure S1. ^{1}H-NMR (DMSO-d_6) spectrum of *N*-(2,5-dimethoxyphenyl)-2-hydroxynaphthalene-1-carboxamide (**5**)

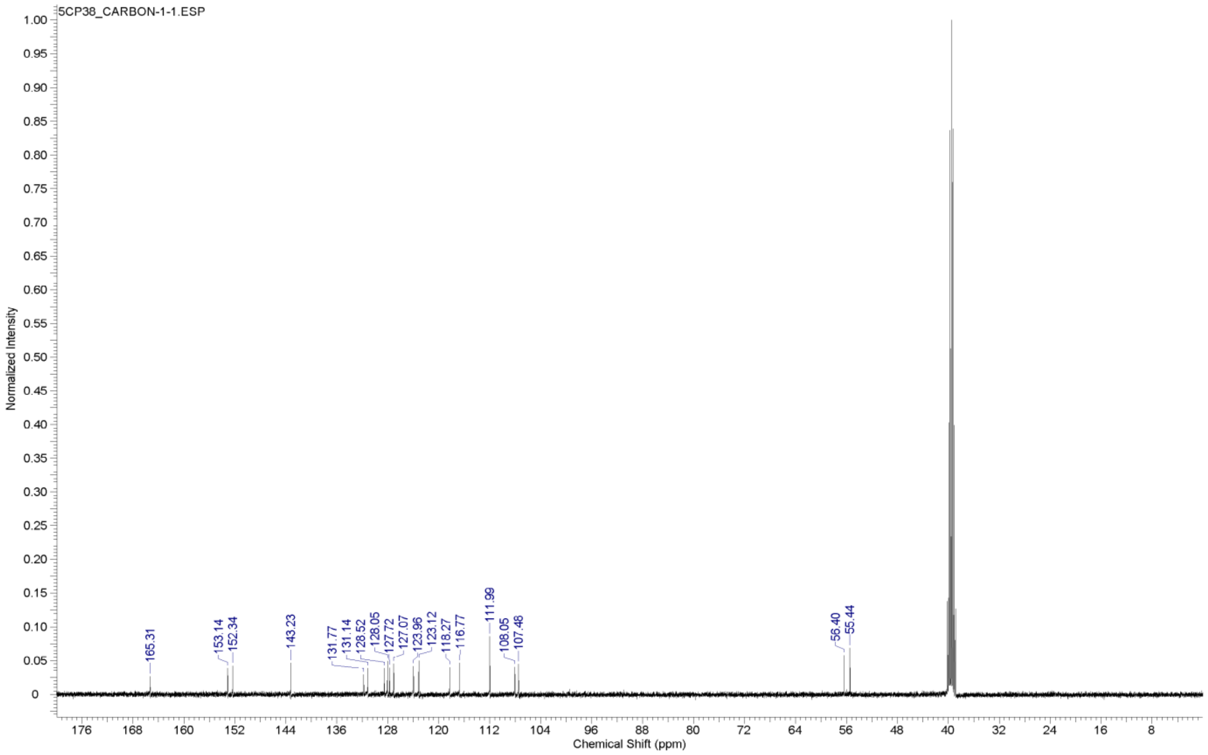

Figure S2. ^{13}C-NMR (DMSO-d_6) spectrum of
N-(2,5-dimethoxyphenyl)-2-hydroxynaphthalene-1-carboxamide (**5**)

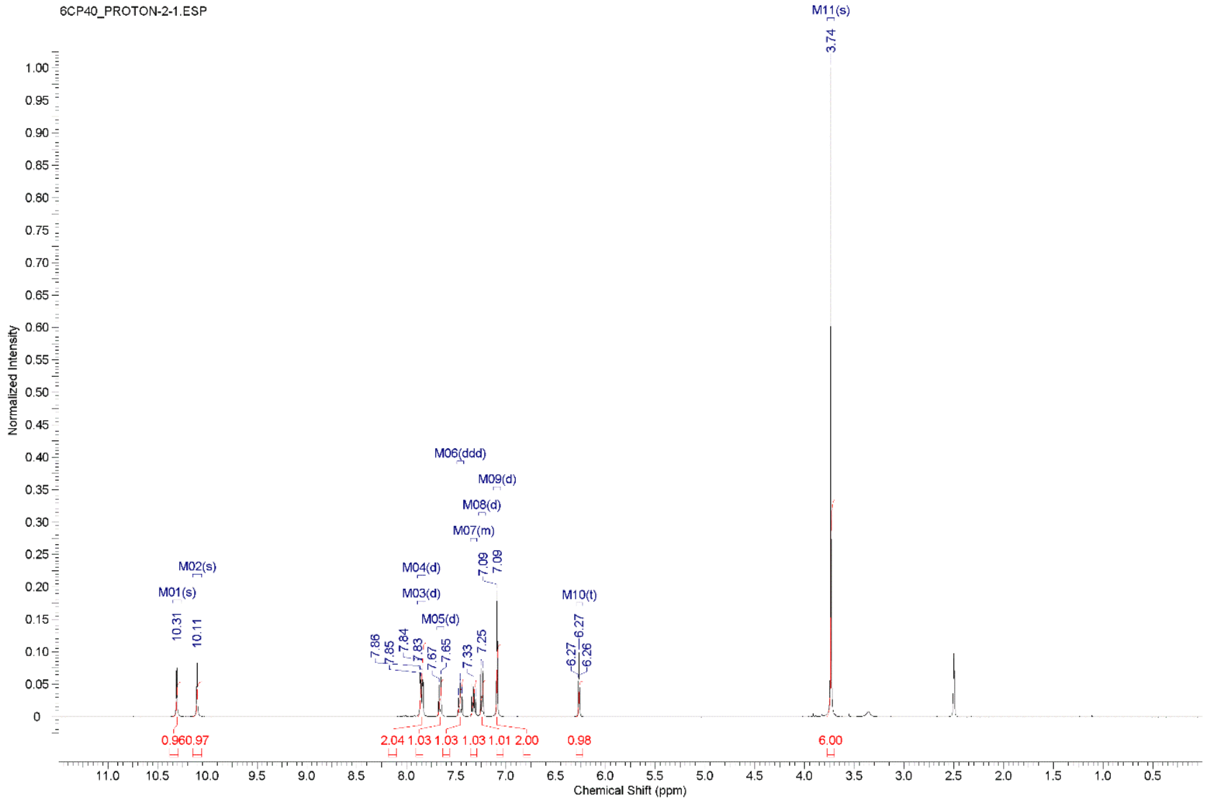

Figure S3. ^{1}H-NMR (DMSO-d_6) spectrum of
N-(3,5-dimethoxyphenyl)-2-hydroxynaphthalene-1-carboxamide (**6**)

CC BY

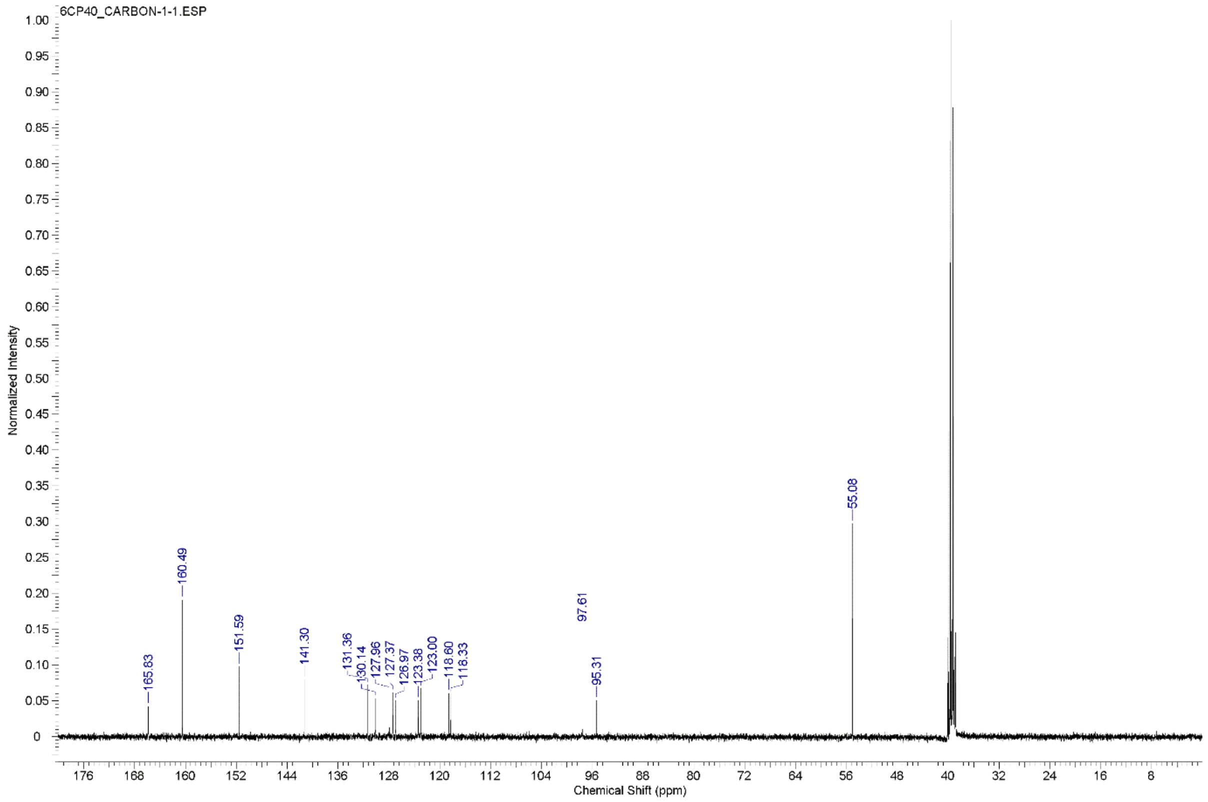

Figure S4. ^{13}C-NMR (DMSO-*d*$_6$) spectrum of *N*-(3,5-dimethoxyphenyl)-2-hydroxynaphthalene-1-carboxamide (**6**)

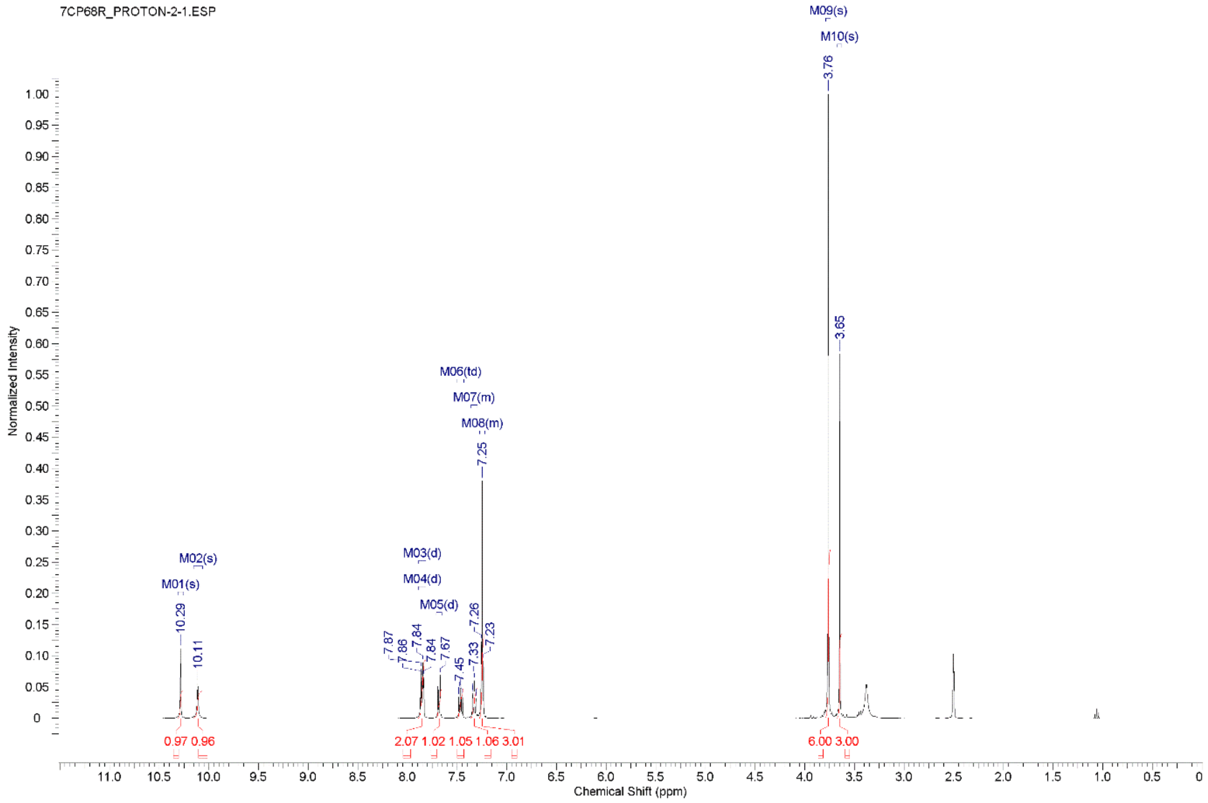

Figure S5. ^{1}H-NMR (DMSO-*d*$_6$) spectrum of 2-hydroxy-*N*-(3,4,5-trimethoxyphenyl)naphthalene-1-carboxamide (**7**)

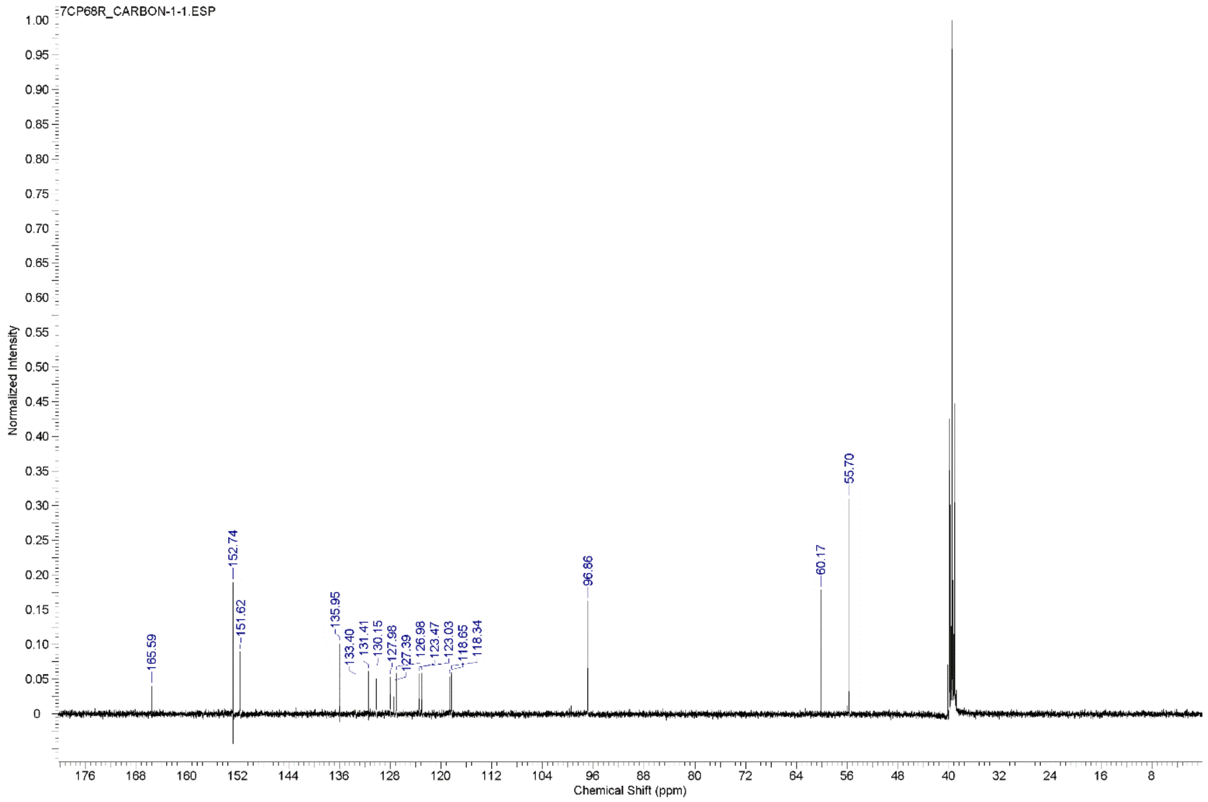

Figure S6. ^{13}C-NMR (DMSO-d_6) spectrum of 2-hydroxy-*N*-(3,4,5-trimethoxyphenyl)naphthalene-1-carboxamide (**7**)

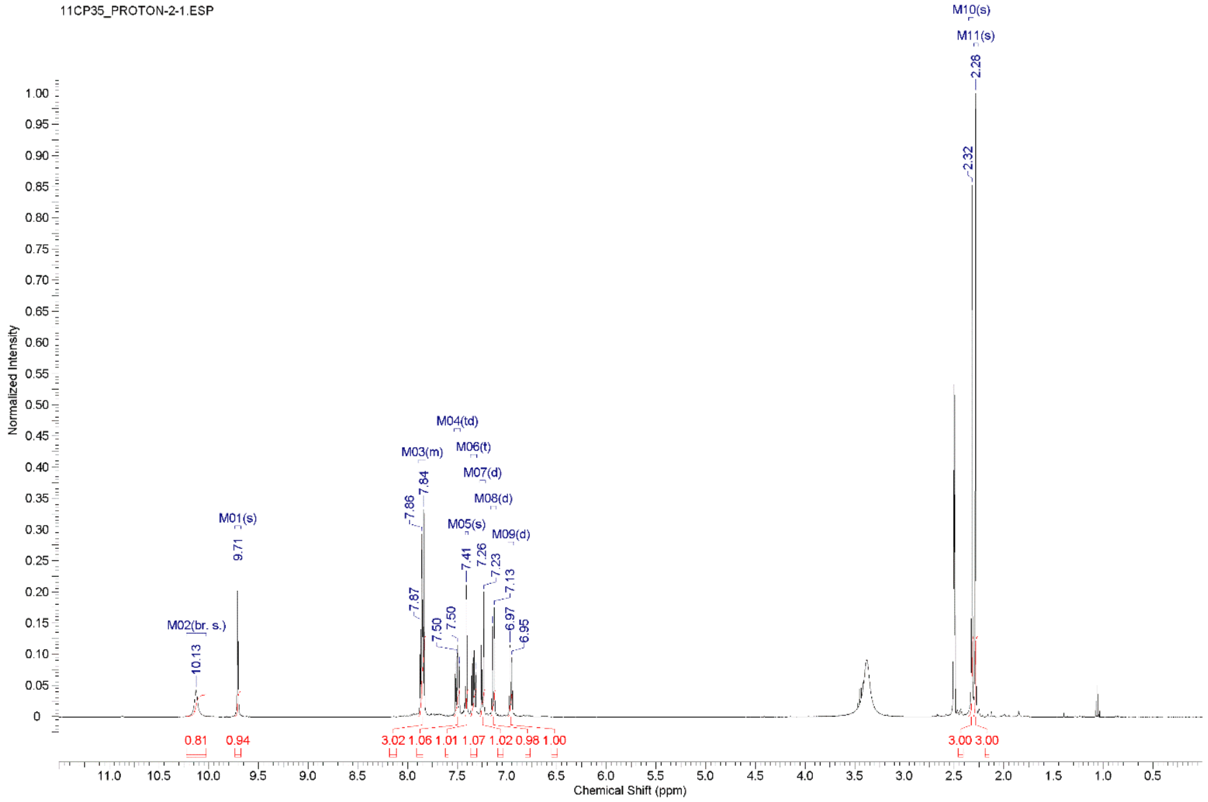

Figure S7. ^{1}H-NMR (DMSO-d_6) spectrum of *N*-(2,5-dimethylphenyl)-2-hydroxynaphthalene-1-carboxamide (**11**)

CC BY

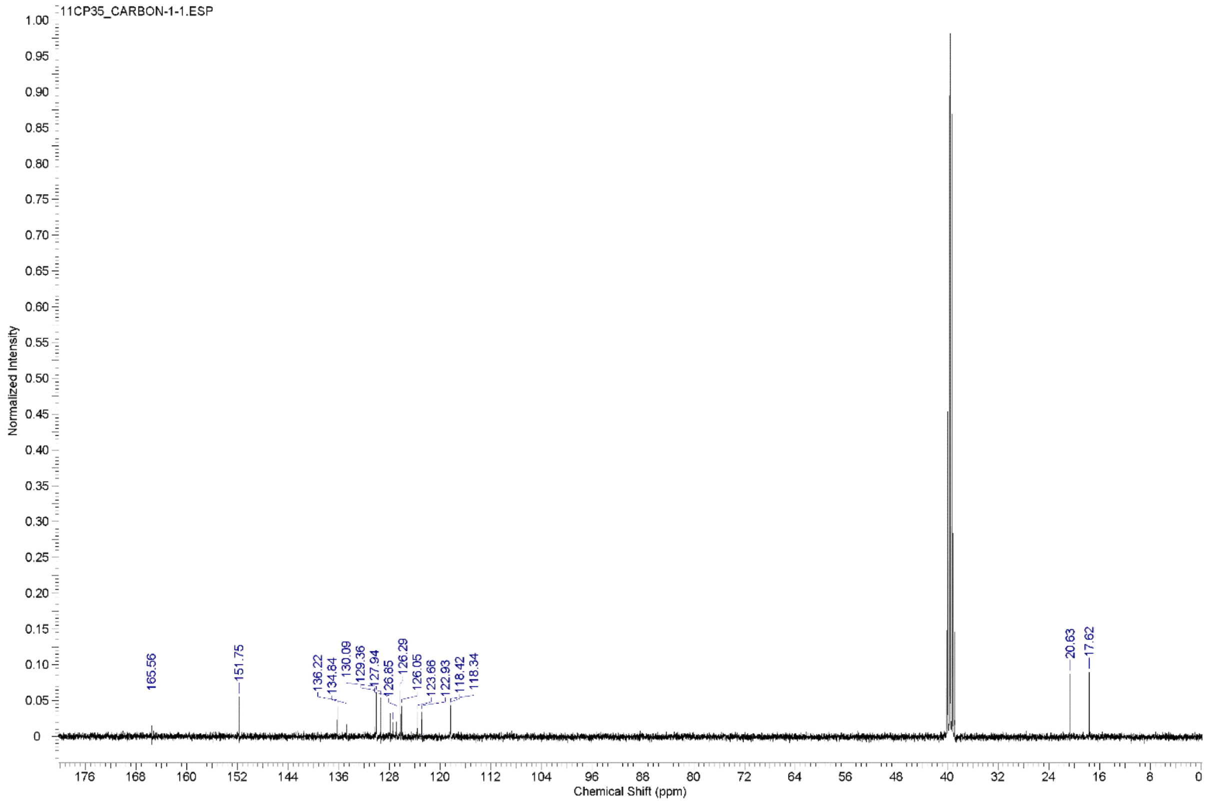

Figure S8. ^{13}C-NMR (DMSO-d_6) spectrum of *N*-(2,5-dimethylphenyl)-2-hydroxynaphthalene-1-carboxamide (**11**)

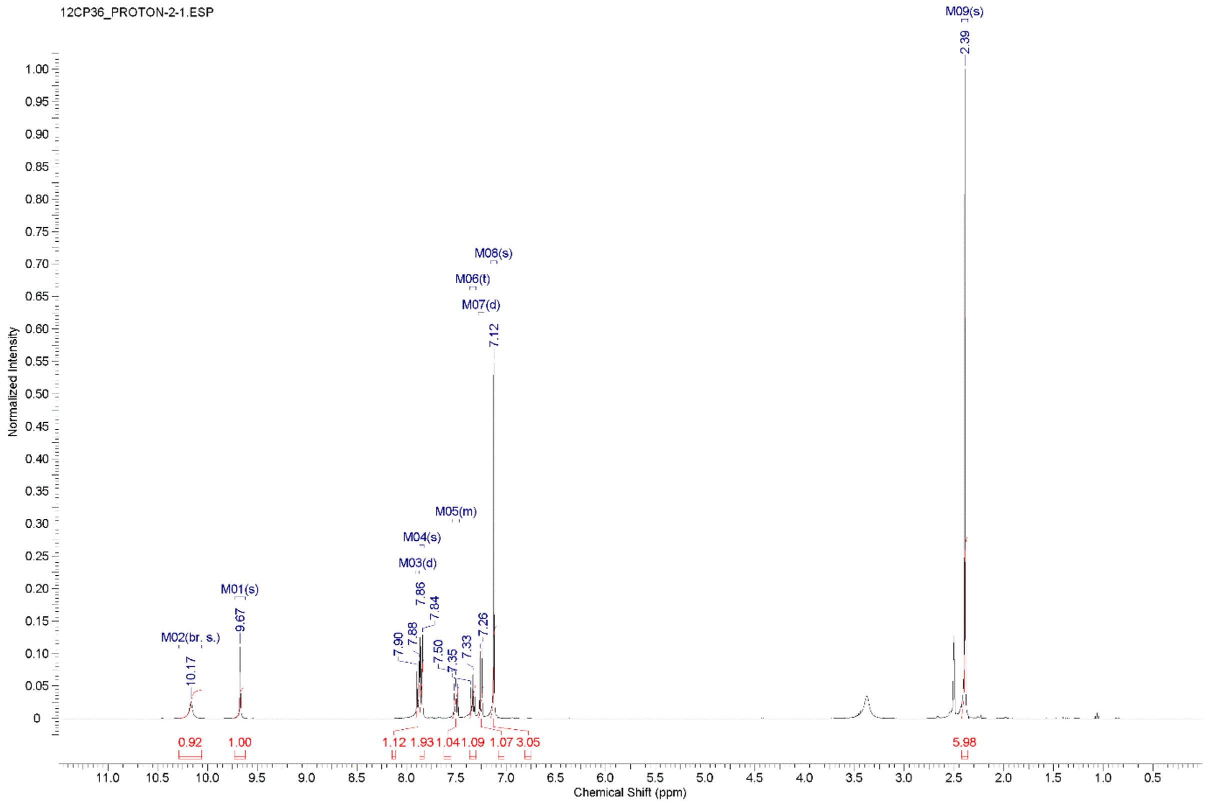

Figure S9. ^{1}H-NMR (DMSO-d_6) spectrum of *N*-(2,6-dimethylphenyl)-2-hydroxynaphthalene-1-carboxamide (**12**)

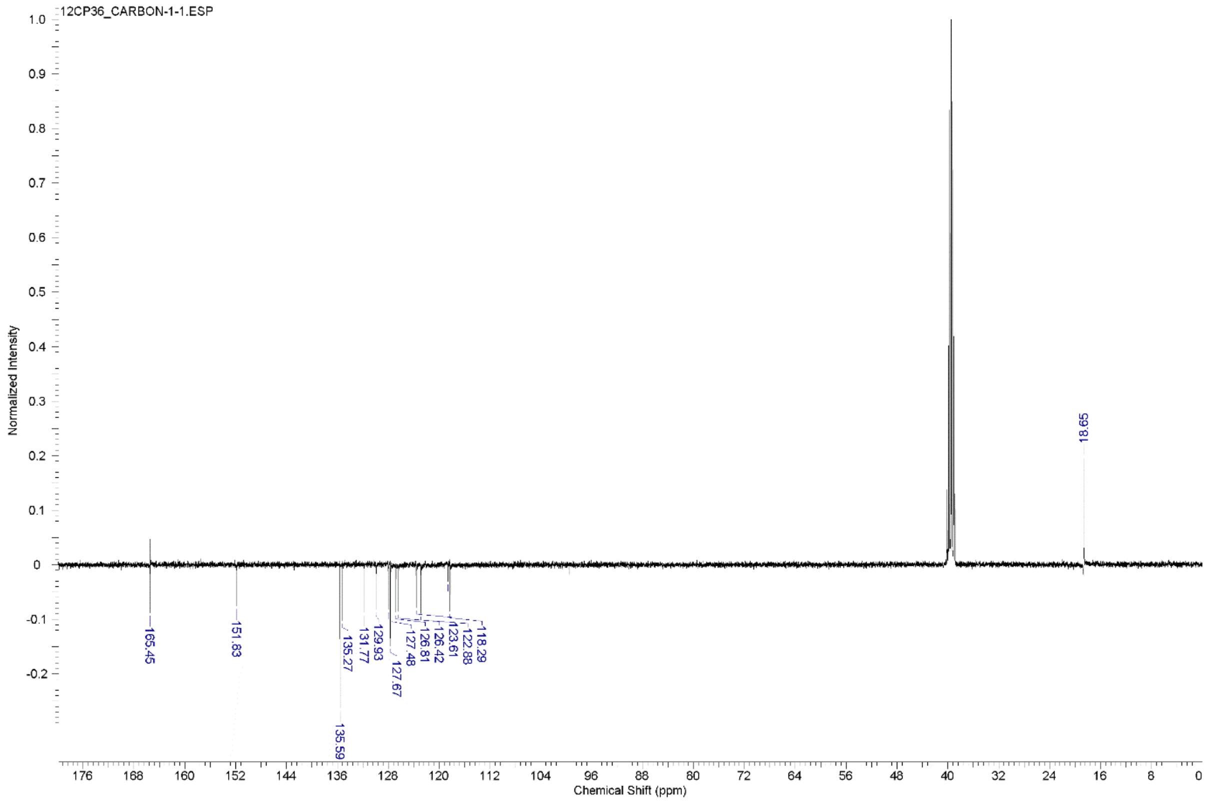

Figure S10. ^{13}C-NMR (DMSO-d_6) spectrum of *N*-(2,6-dimethylphenyl)-2-hydroxynaphthalene-1-carboxamide (**12**)

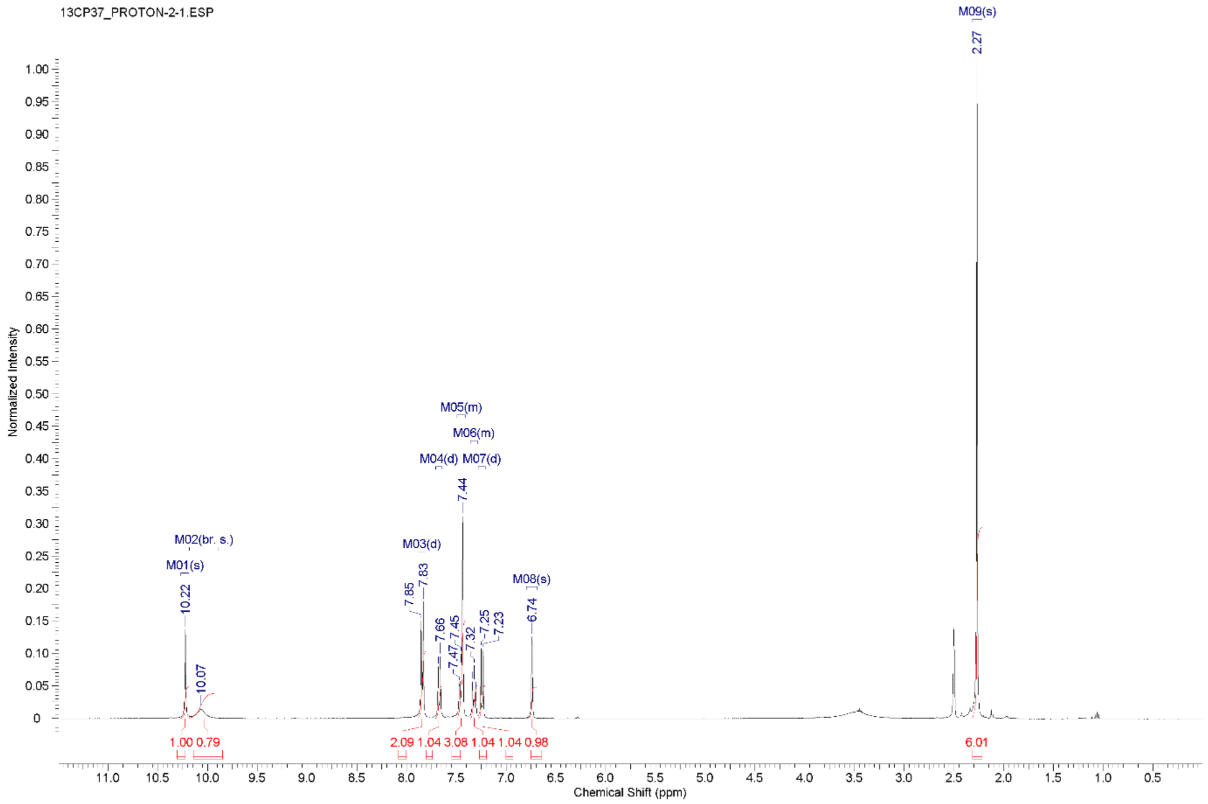

Figure S11. ^{1}H-NMR (DMSO-d_6) spectrum of *N*-(3,5-dimethylphenyl)-2-hydroxynaphthalene-1-carboxamide (**13**)

CC BY

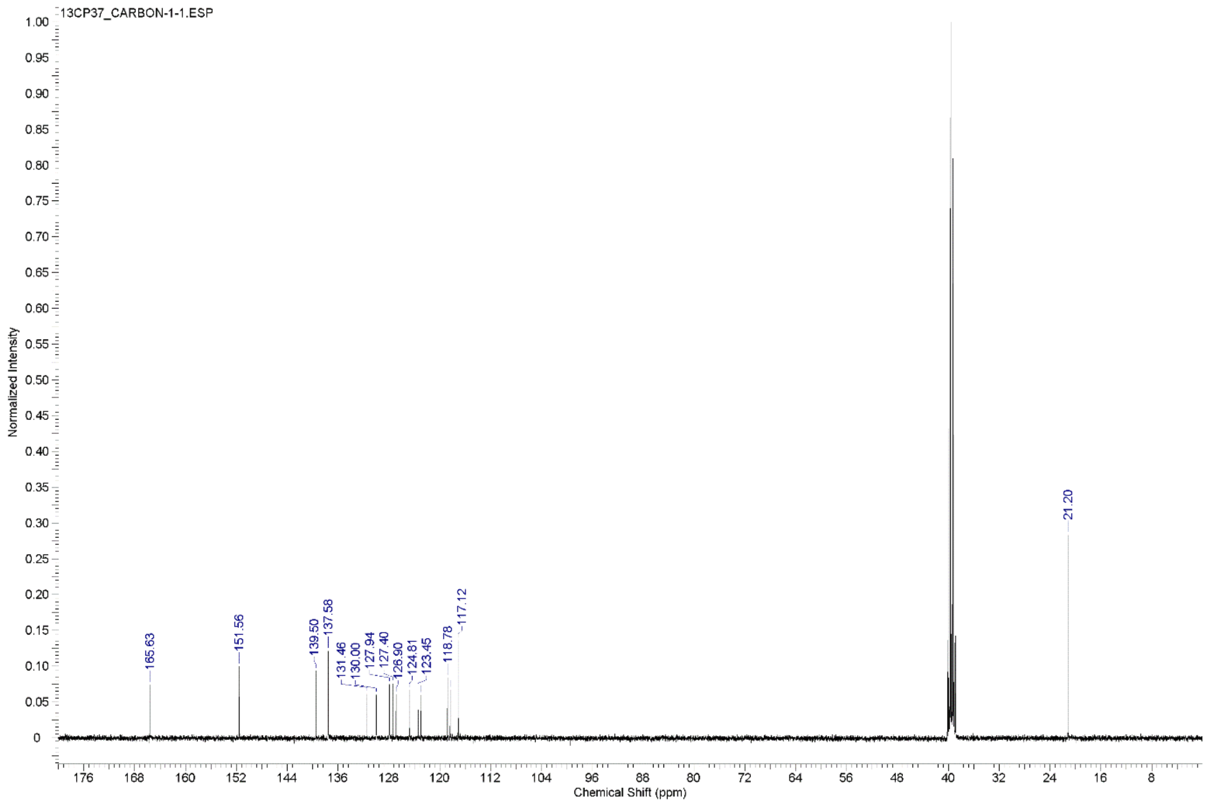

Figure S12. ^{13}C-NMR (DMSO-d_6) spectrum of
N-(3,5-dimethylphenyl)-2-hydroxynaphthalene-1-carboxamide (**13**)

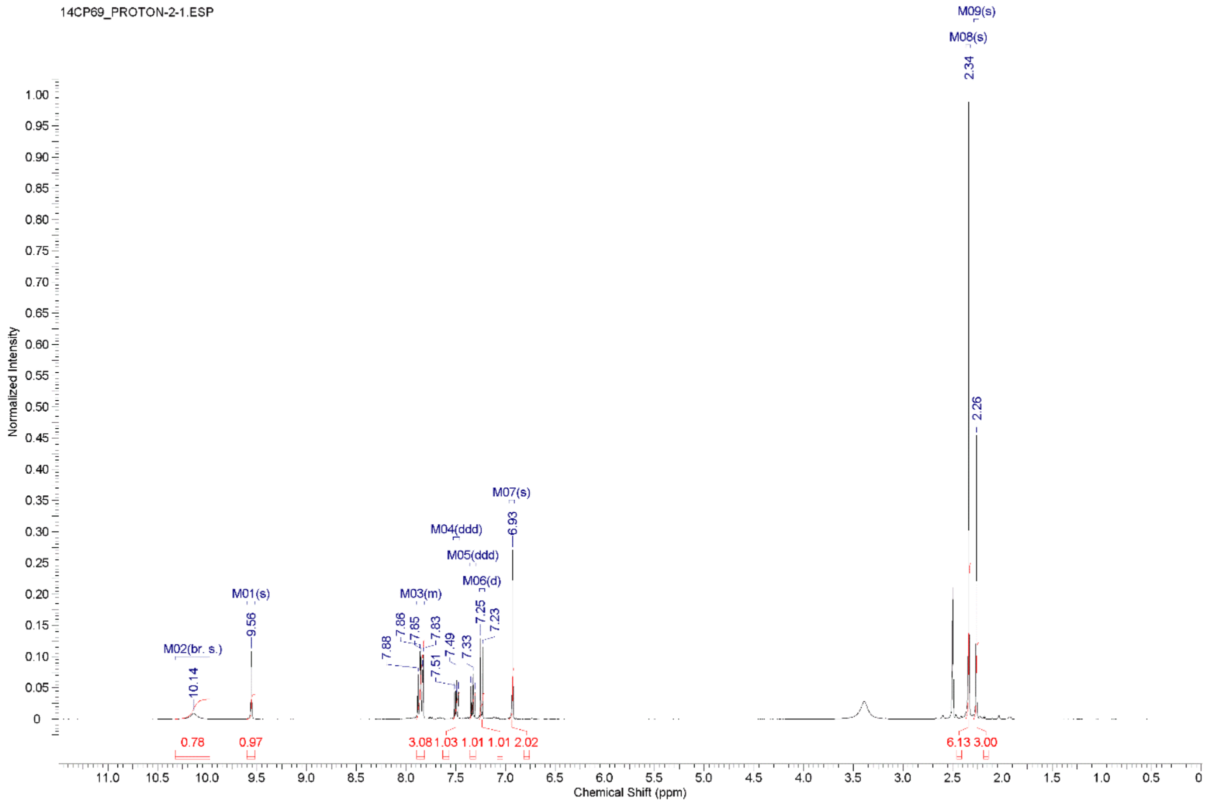

Figure S13. ^{1}H-NMR (DMSO-d_6) spectrum of
2-hydroxy-*N*-(2,4,6-trimethylphenyl)naphthalene-1-carboxamide (**14**)

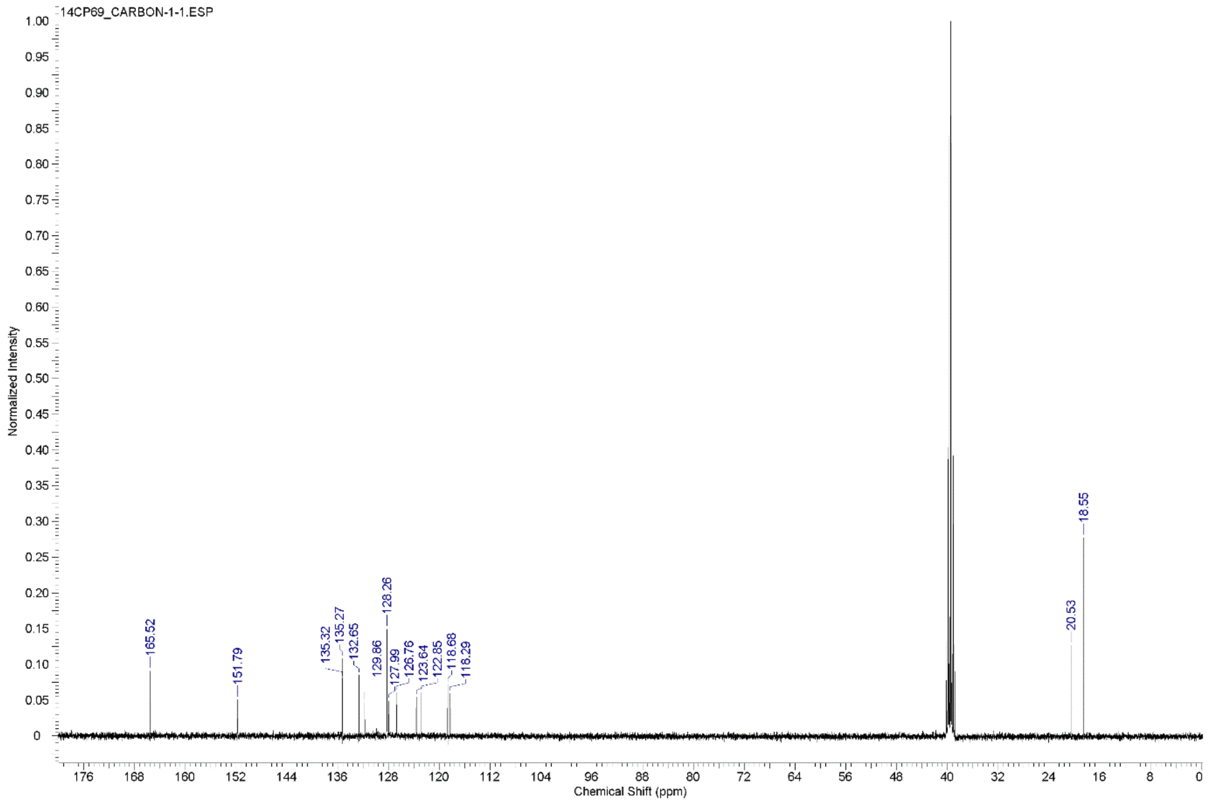

Figure S14. ^{13}C-NMR (DMSO-d_6) spectrum of 2-hydroxy-*N*-(2,4,6-trimethylphenyl)naphthalene-1-carboxamide (**14**)

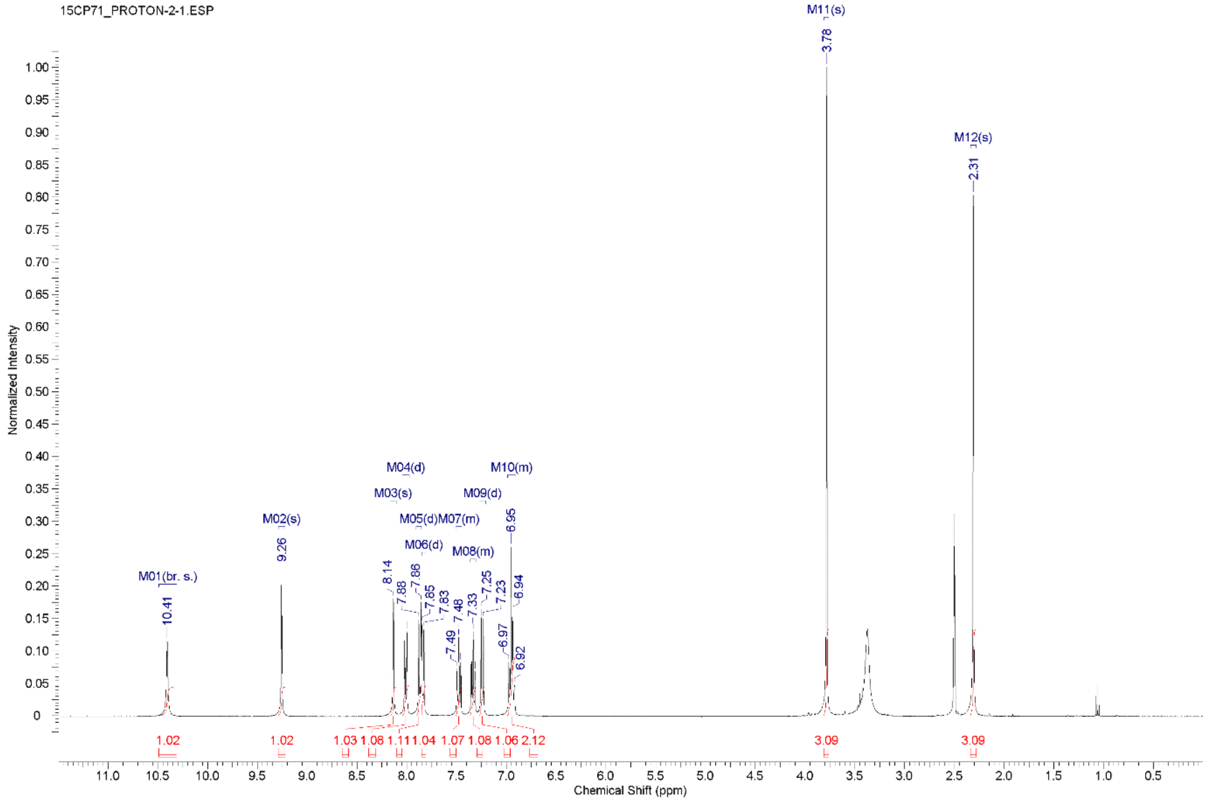

Figure S15. ^{1}H-NMR (DMSO-d_6) spectrum of 2-hydroxy-*N*-(2-methoxy-5-methylphenyl)naphthalene-1-carboxamide (**15**)

CC BY

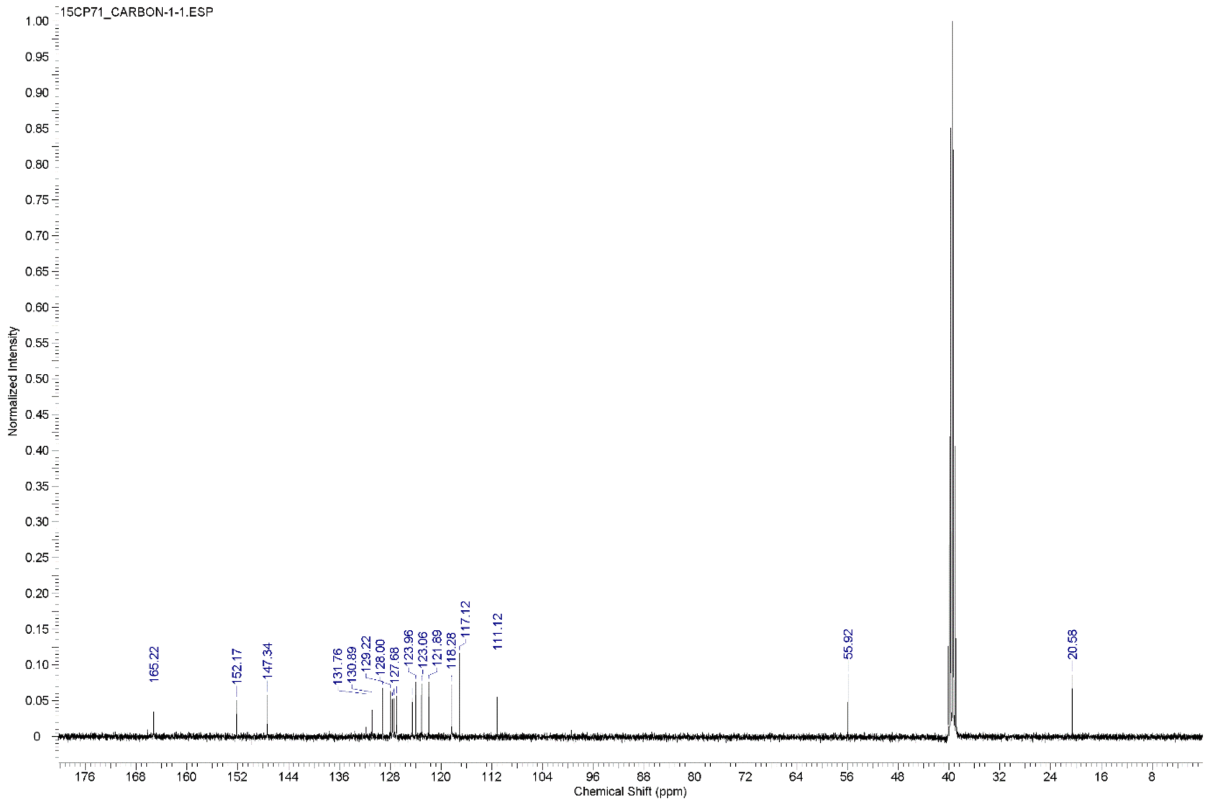

Figure S16. ^{13}C-NMR (DMSO-d_6) spectrum of 2-hydroxy-*N*-(2-methoxy-5-methylphenyl)naphthalene-1-carboxamide (**15**)

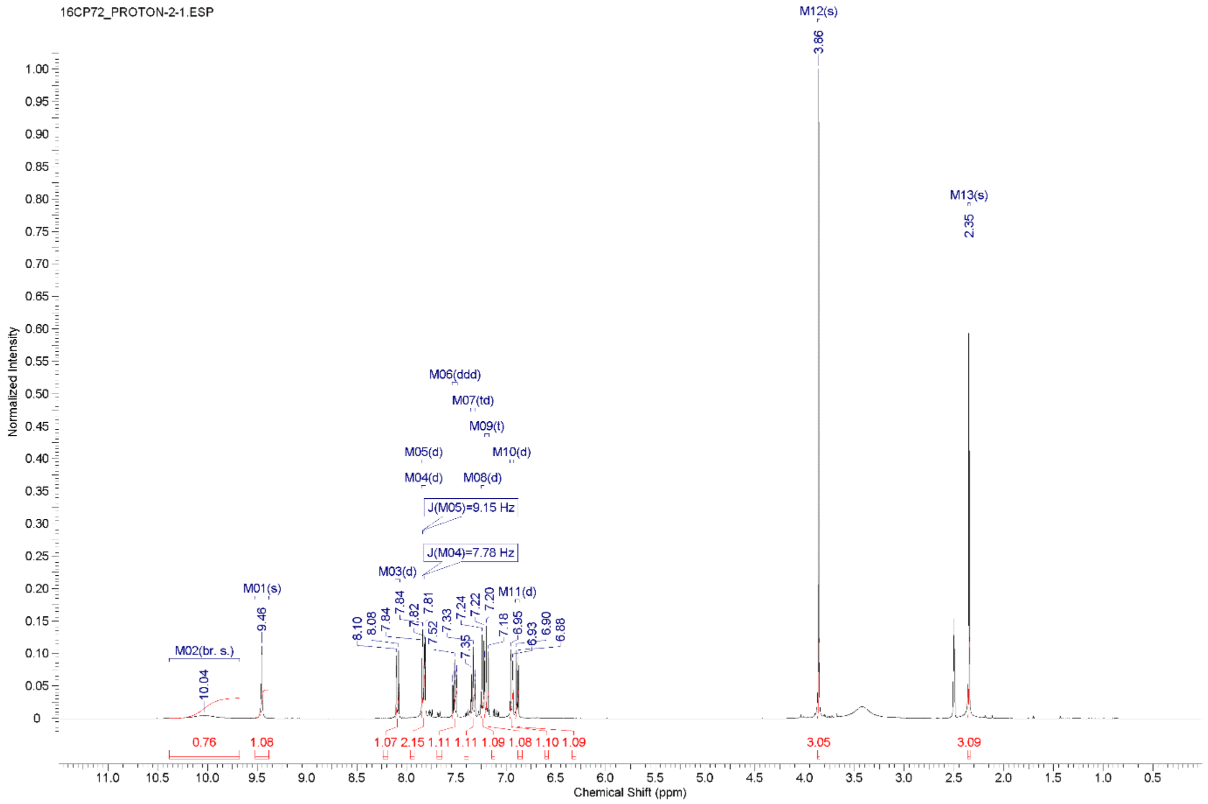

Figure S17. ^{1}H-NMR (DMSO-d_6) spectrum of 2-hydroxy-*N*-(2-methoxy-6-methylphenyl)naphthalene-1-carboxamide (**16**)

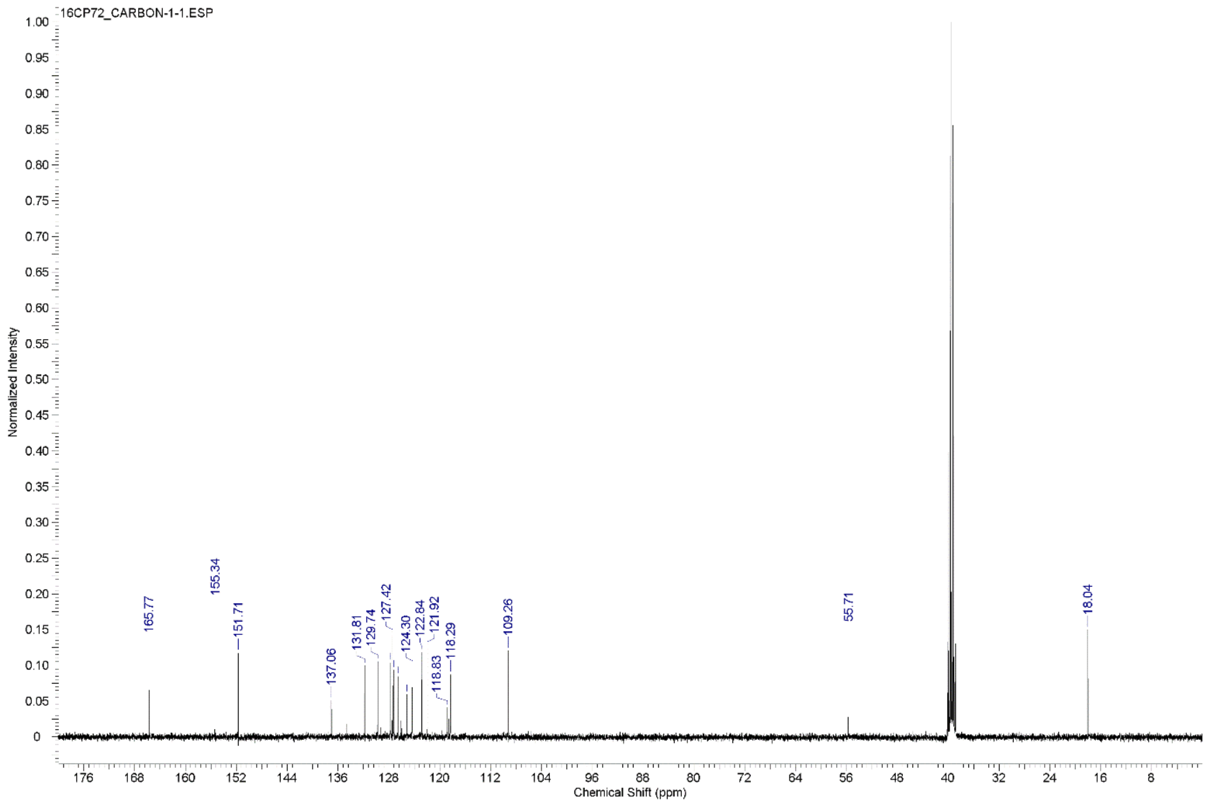

Figure S18. ^{13}C-NMR (DMSO-d_6) spectrum of 2-hydroxy-*N*-(2-methoxy-6-methylphenyl)naphthalene-1-carboxamide (**16**)

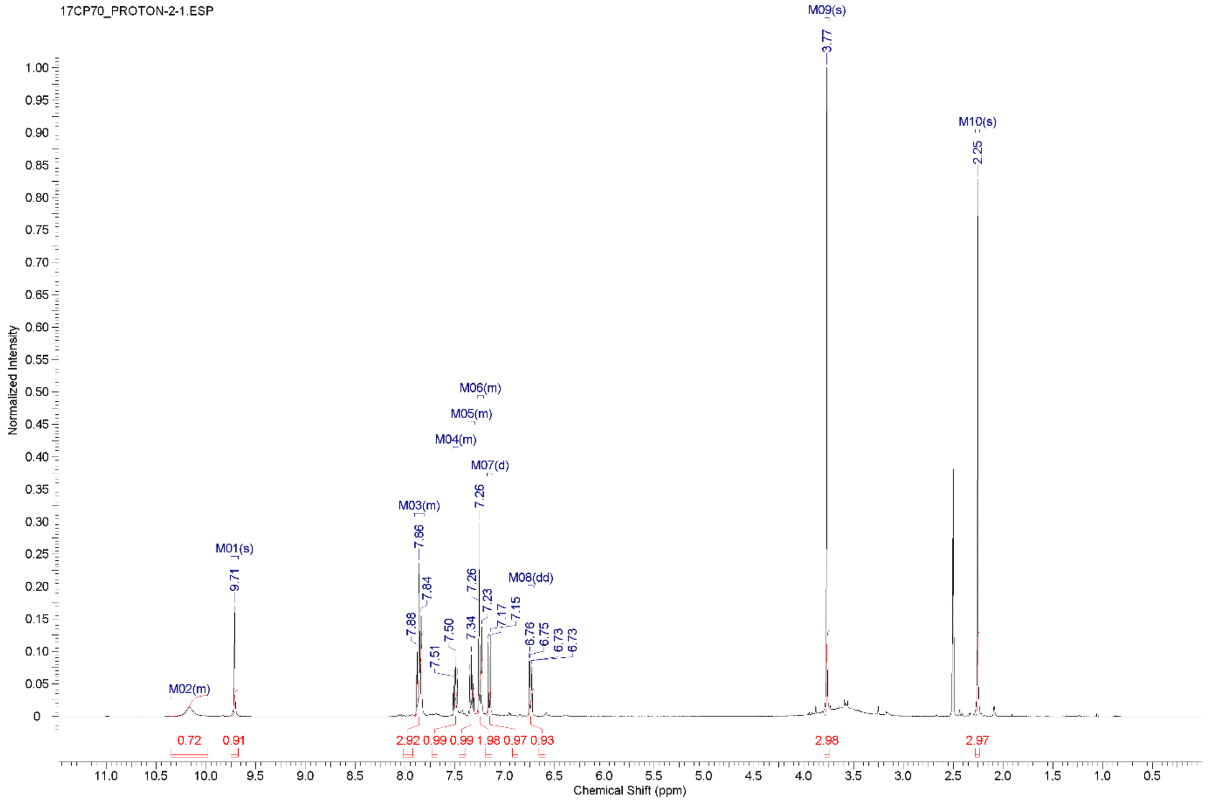

Figure S19. ^{1}H-NMR (DMSO-d_6) spectrum of 2-hydroxy-*N*-(5-methoxy-2-methylphenyl)naphthalene-1-carboxamide (**17**)

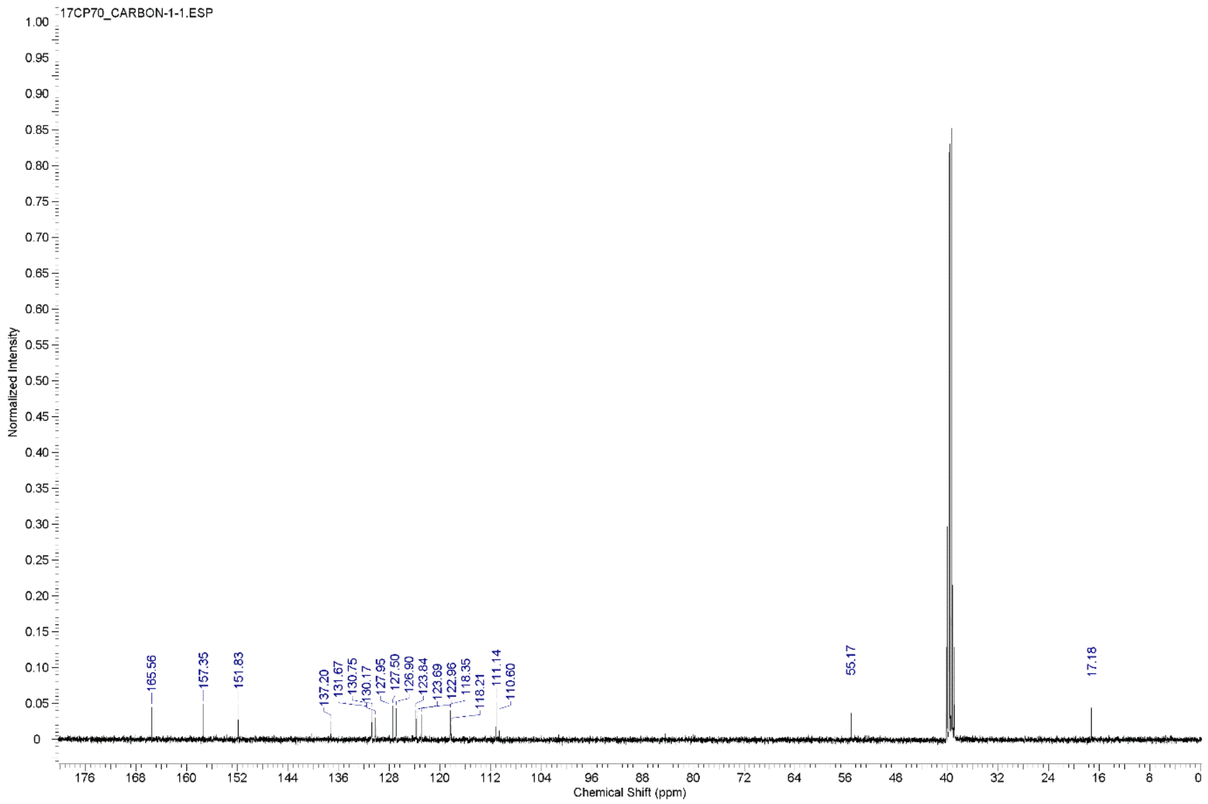

Figure S20. ^{13}C-NMR (DMSO-*d*$_6$) spectrum of
2-hydroxy-*N*-(5-methoxy-2-methylphenyl)naphthalene-1-carboxamide (**17**)

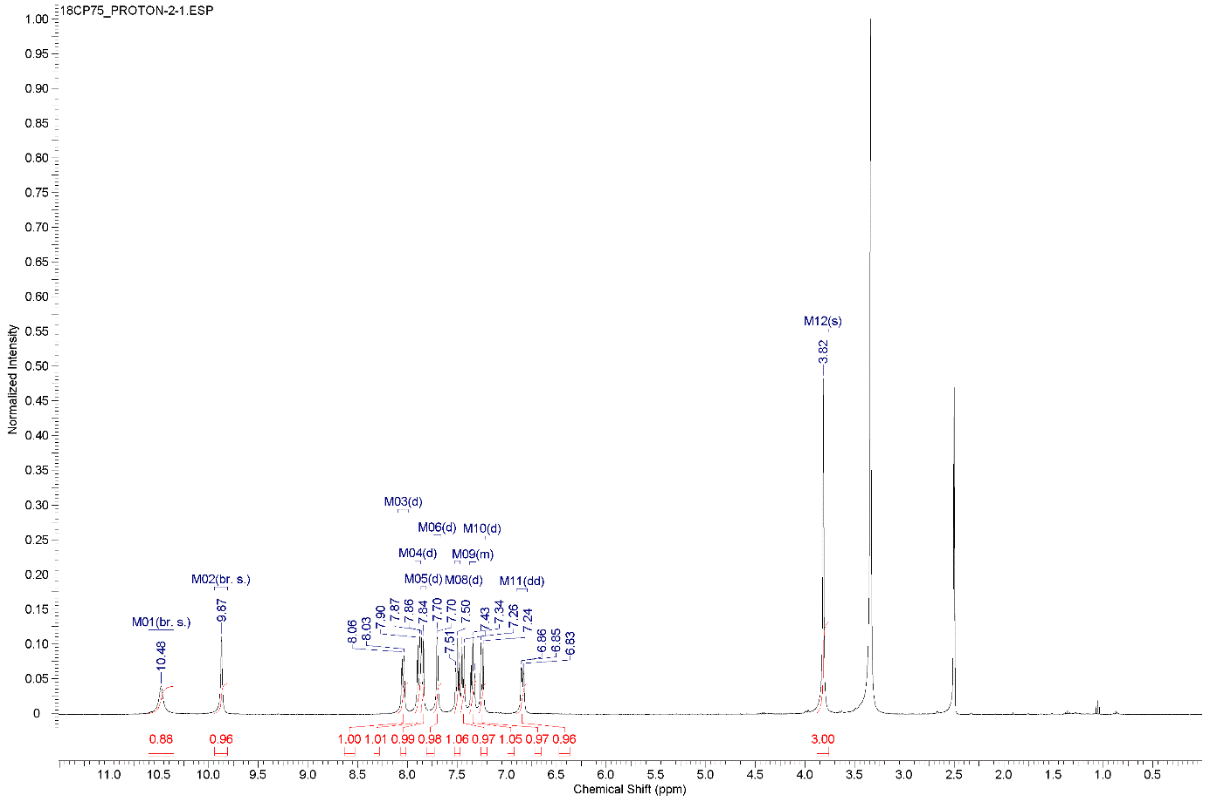

Figure S21. ^{1}H-NMR (DMSO-*d*$_6$) spectrum of
N-(2-chloro-5-methoxyphenyl)-2-hydroxynaphthalene-1-carboxamide (**18**)

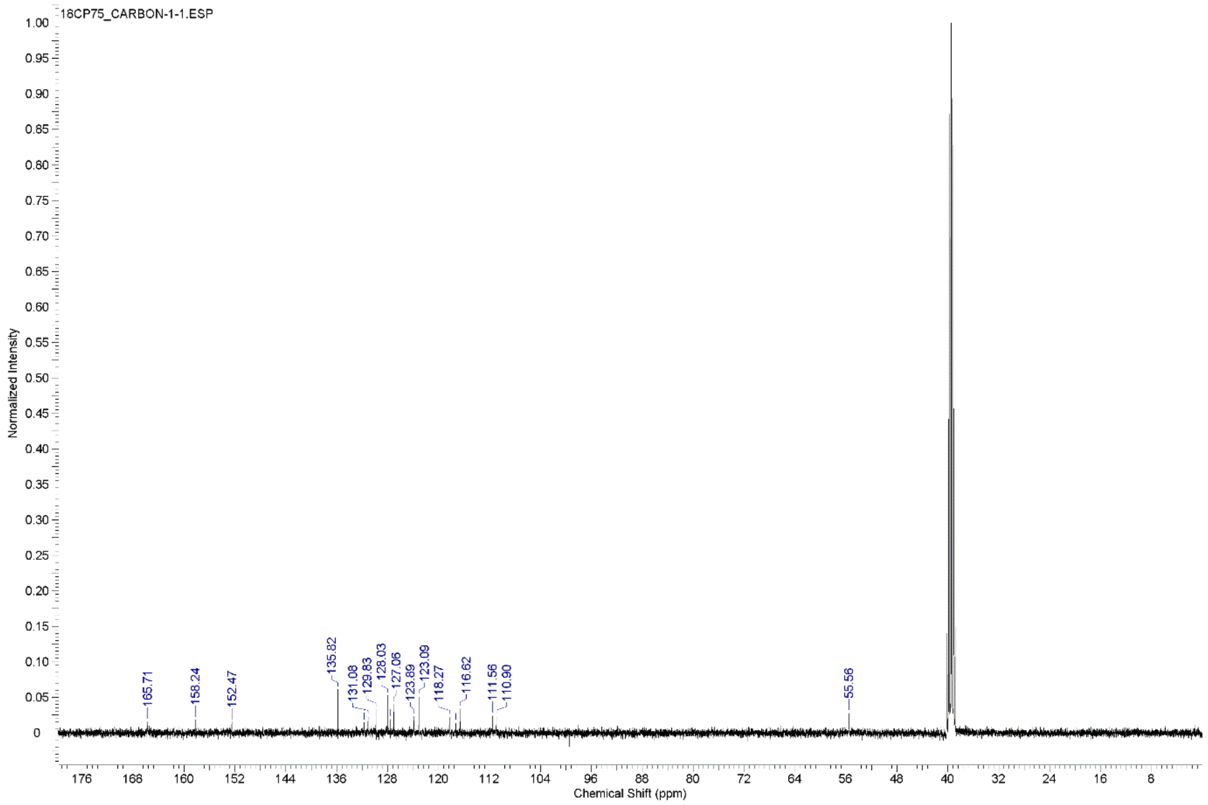

Figure S22. ^{13}C-NMR (DMSO-d_6) spectrum of *N*-(2-chloro-5-methoxyphenyl)-2-hydroxynaphthalene-1-carboxamide (**18**)

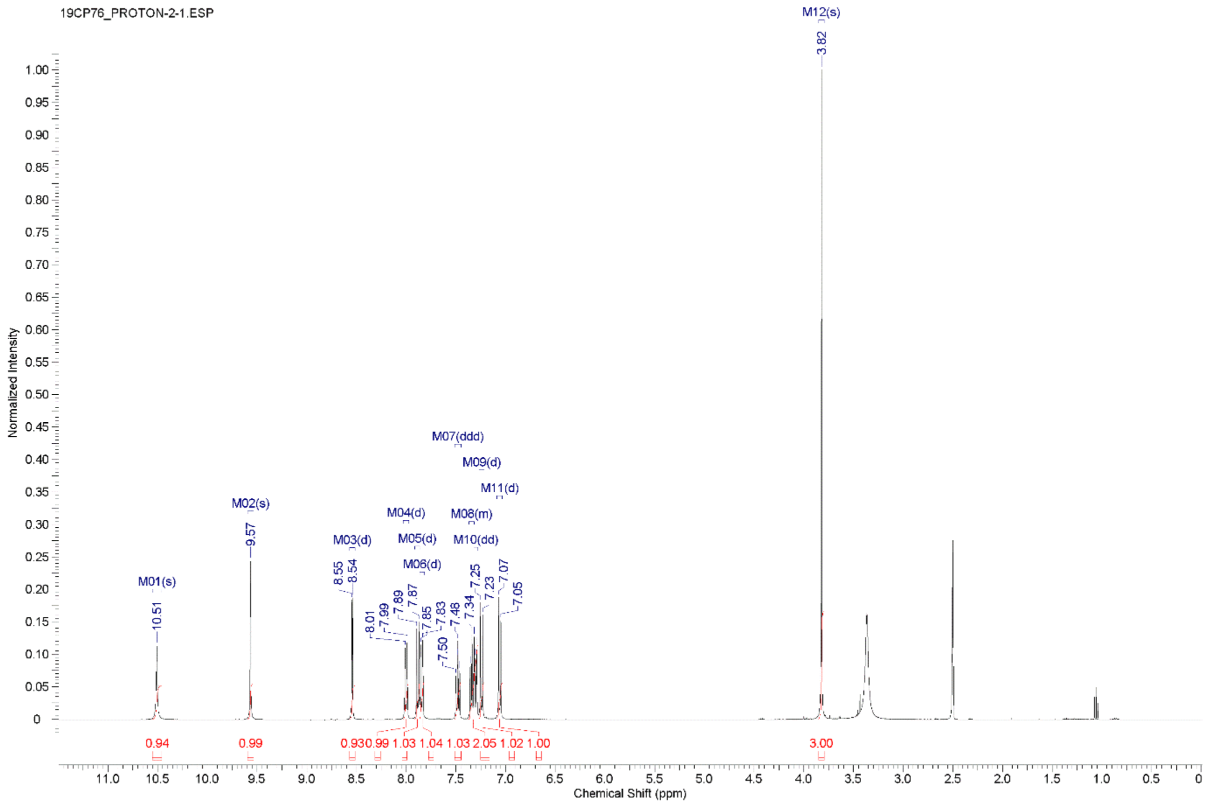

Figure S23. ^{1}H-NMR (DMSO-d_6) spectrum of *N*-(5-bromo-2-methoxyphenyl)-2-hydroxynaphthalene-1-carboxamide (**19**)

CC BY

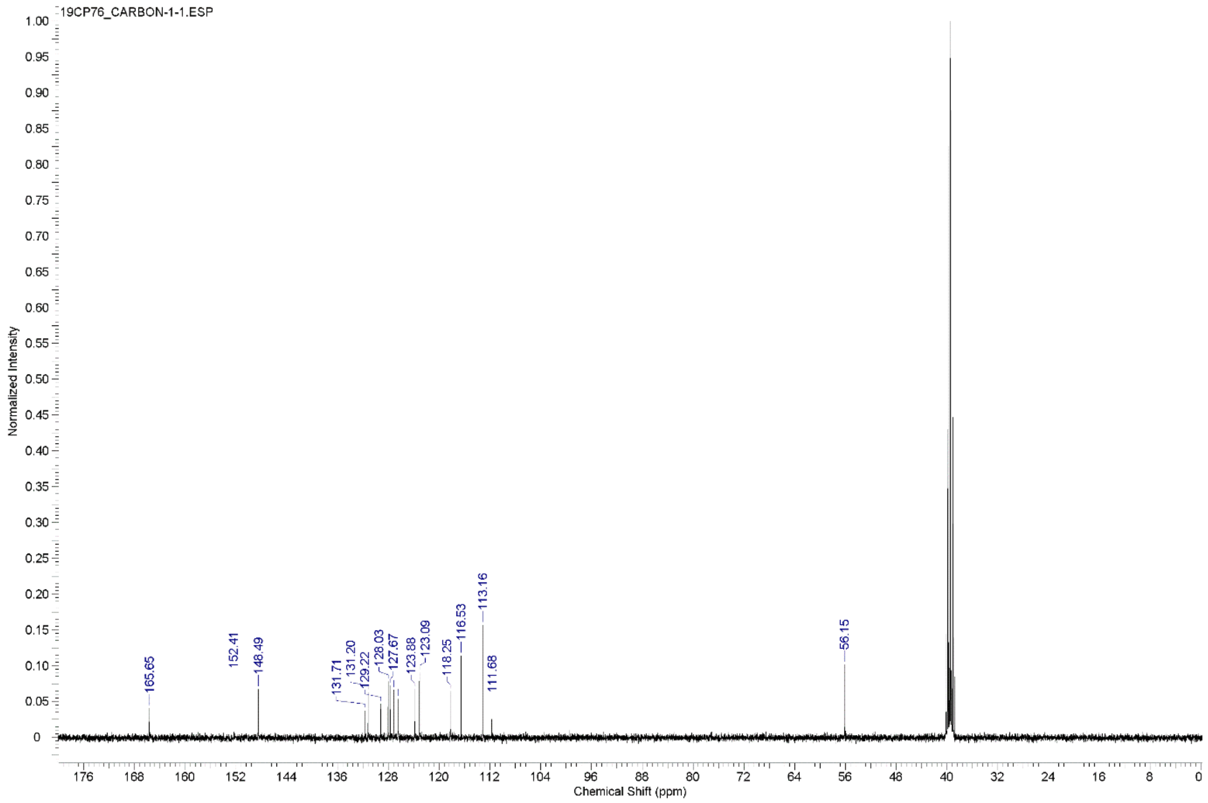

Figure S24. ^{13}C-NMR (DMSO-d_6) spectrum of
N-(5-bromo-2-methoxyphenyl)-2-hydroxynaphthalene-1-carboxamide (**19**)

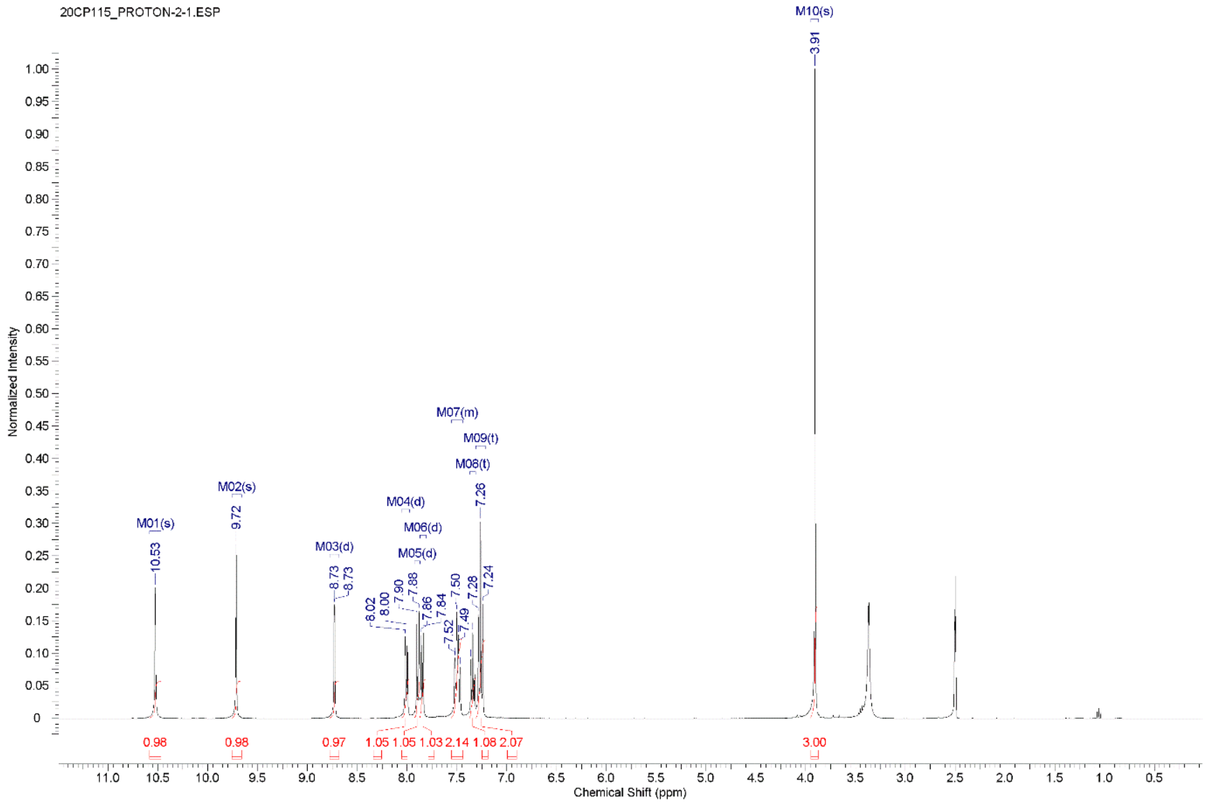

Figure S25. ^{1}H-NMR (DMSO-d_6) spectrum of
2-hydroxy-*N*-[2-methoxy-5-(trifluoromethyl)phenyl]naphthalene-1-carboxamide (**20**)

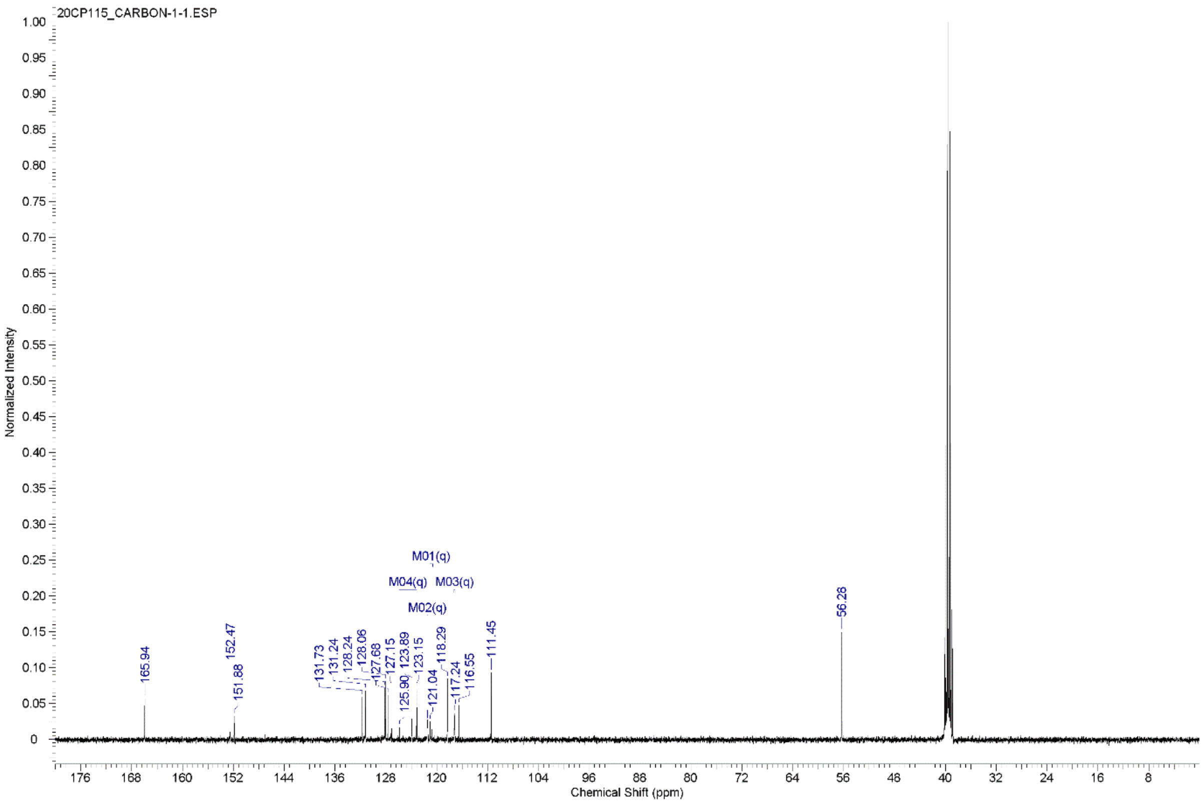

Figure S26. ^{13}C-NMR (DMSO-d_6) spectrum of 2-hydroxy-*N*-[2-methoxy-5-(trifluoromethyl)phenyl]naphthalene-1-carboxamide (**20**)

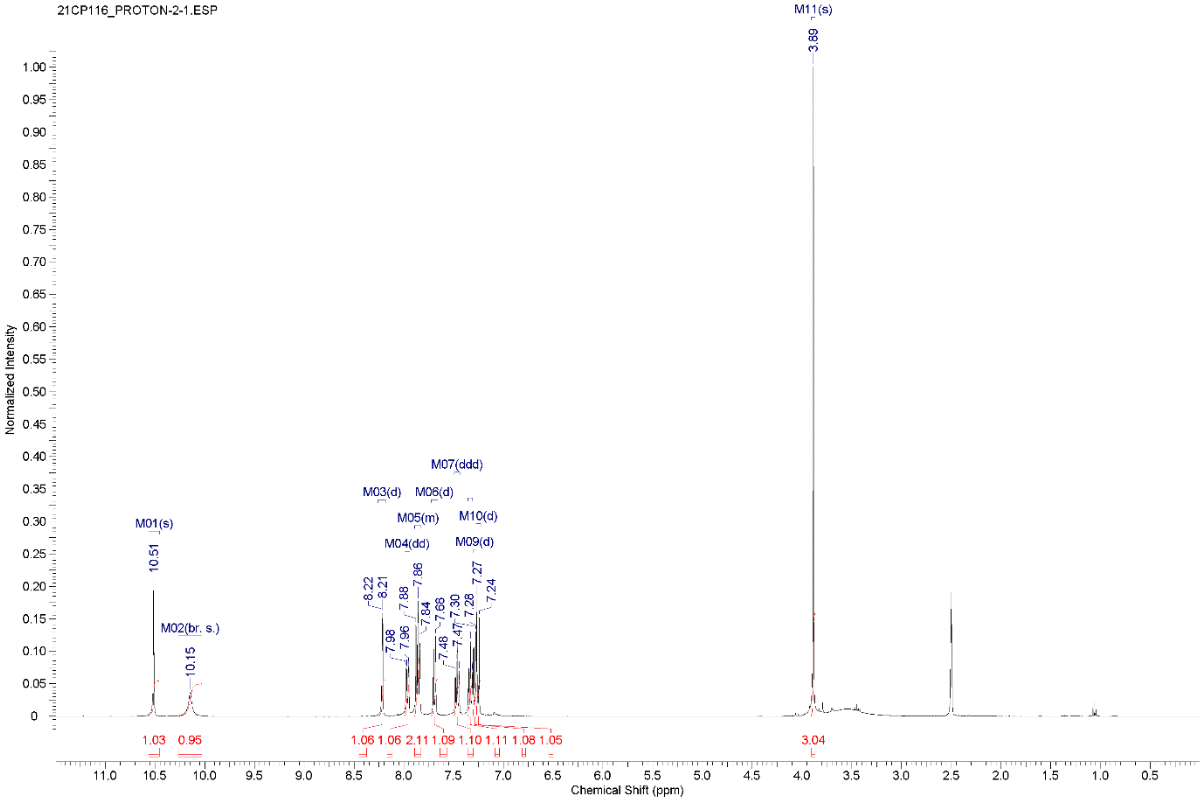

Figure S27. ^{1}H-NMR (DMSO-d_6) spectrum of 2-hydroxy-*N*-[4-methoxy-3-(trifluoromethyl)phenyl]naphthalene-1-carboxamide (**21**).

CC BY

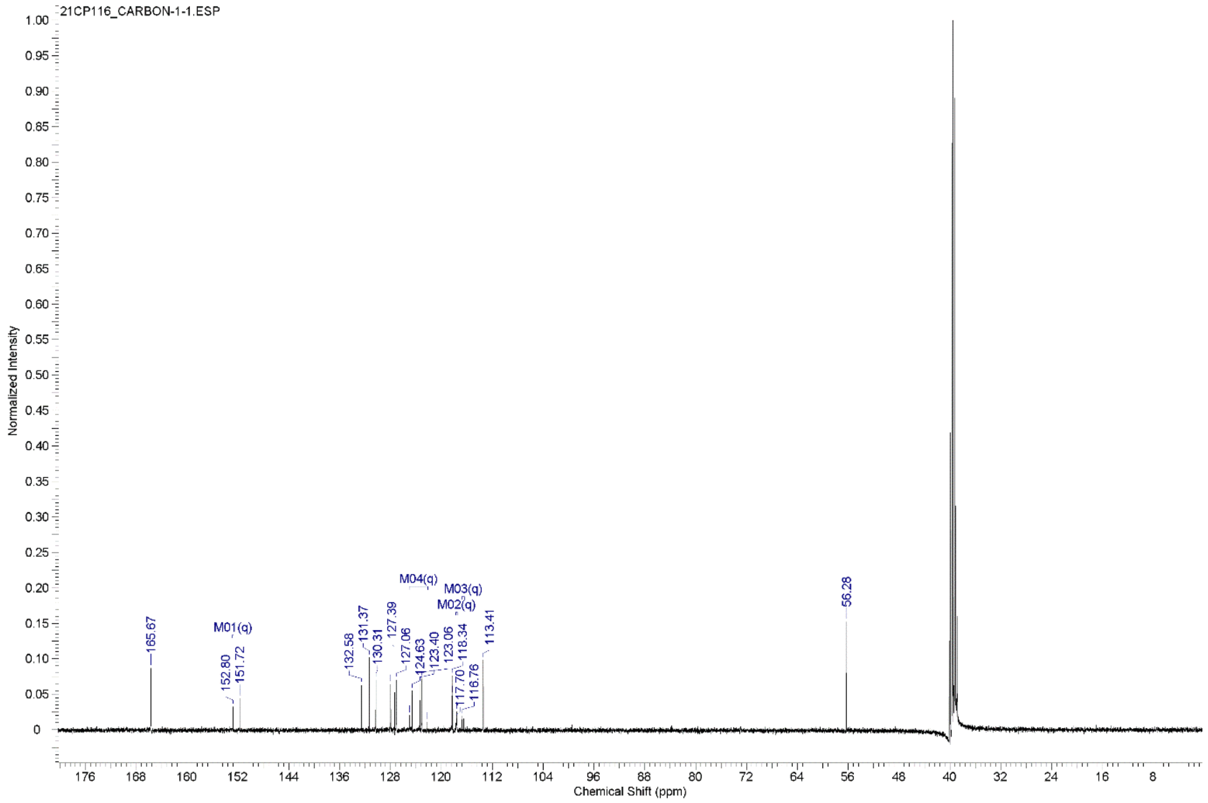

Figure S28. ^{13}C-NMR (DMSO-d_6) spectrum of
2-hydroxy-*N*-[4-methoxy-3-(trifluoromethyl)phenyl]naphthalene-1-carboxamide (**21**)

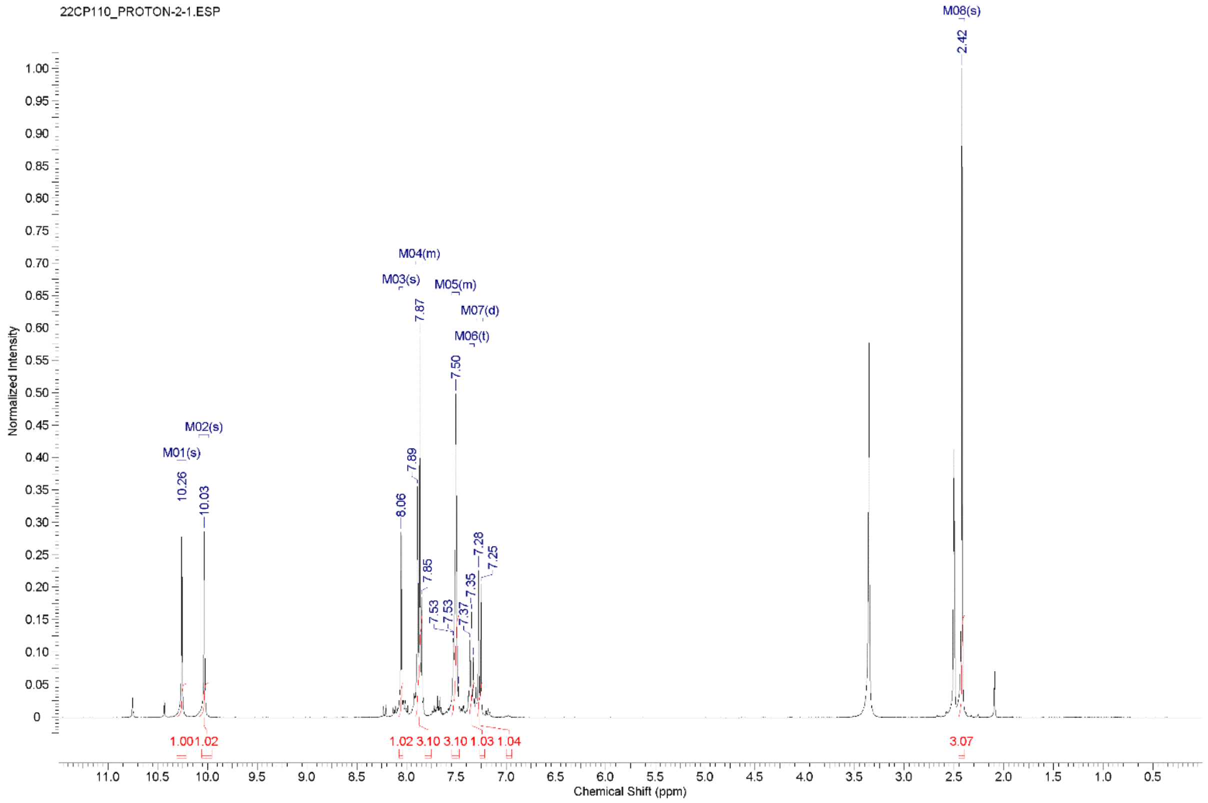

Figure S29. ^{1}H-NMR (DMSO-d_6) spectrum of
2-hydroxy-*N*-[2-methyl-5-(trifluoromethyl)phenyl]naphthalene-1-carboxamide (**22**)

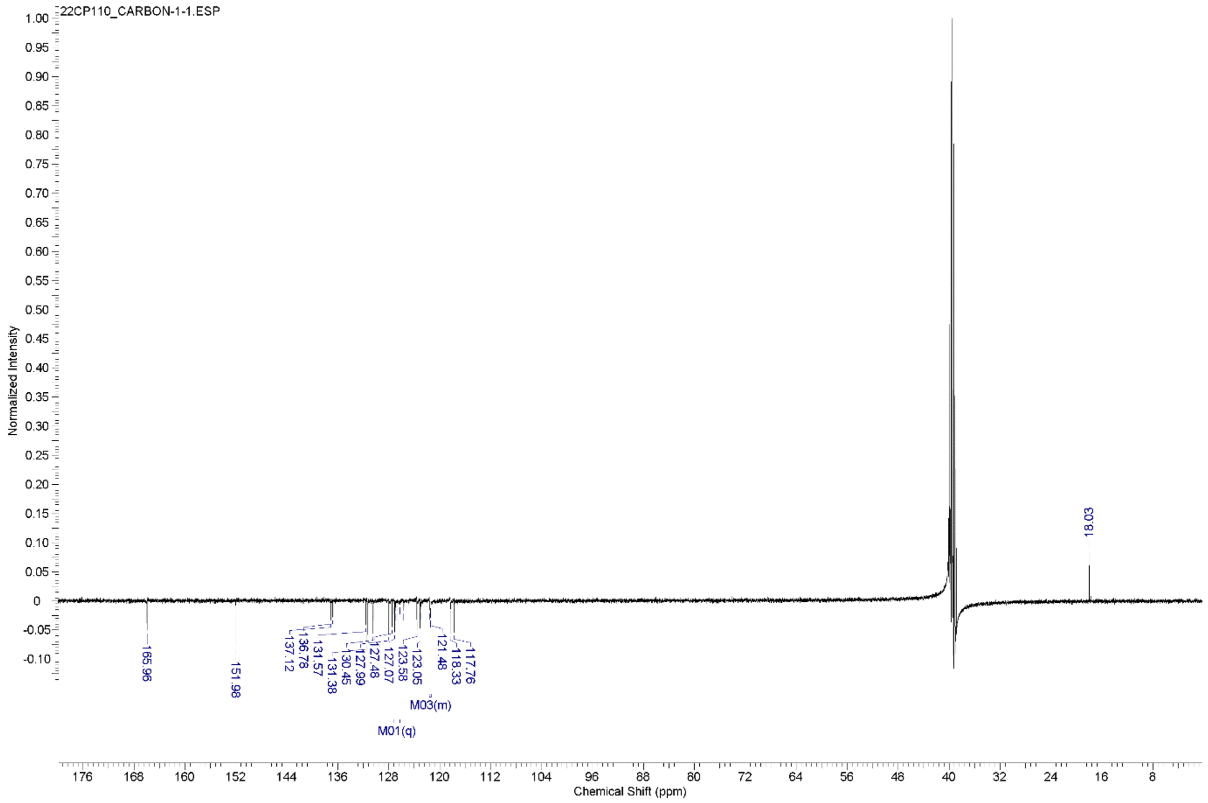

Figure S30. ^{13}C-NMR (DMSO-d_6) spectrum of 2-hydroxy-*N*-[2-methyl-5-(trifluoromethyl)phenyl]naphthalene-1-carboxamide (**22**)

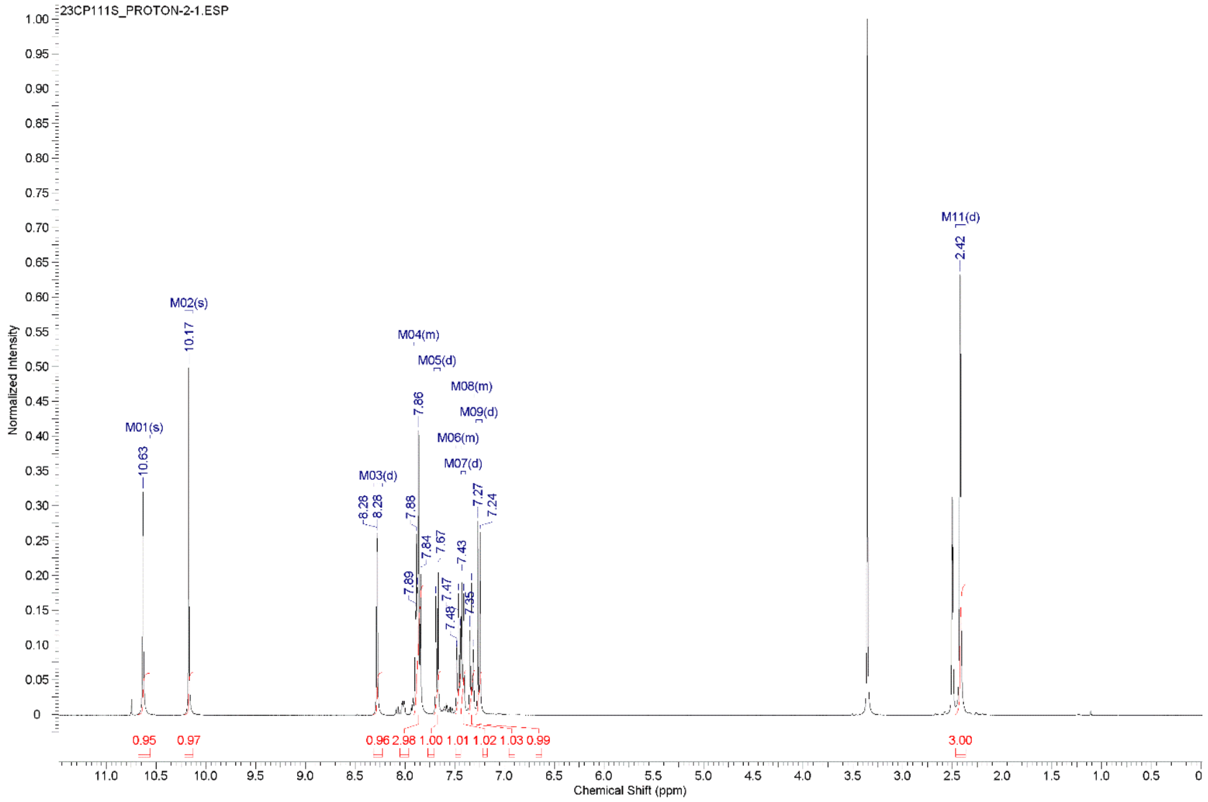

Figure S31. ^{1}H-NMR (DMSO-d_6) spectrum of 2-hydroxy-*N*-[4-methyl-3-(trifluoromethyl)phenyl]naphthalene-1-carboxamide (**23**)

CC BY

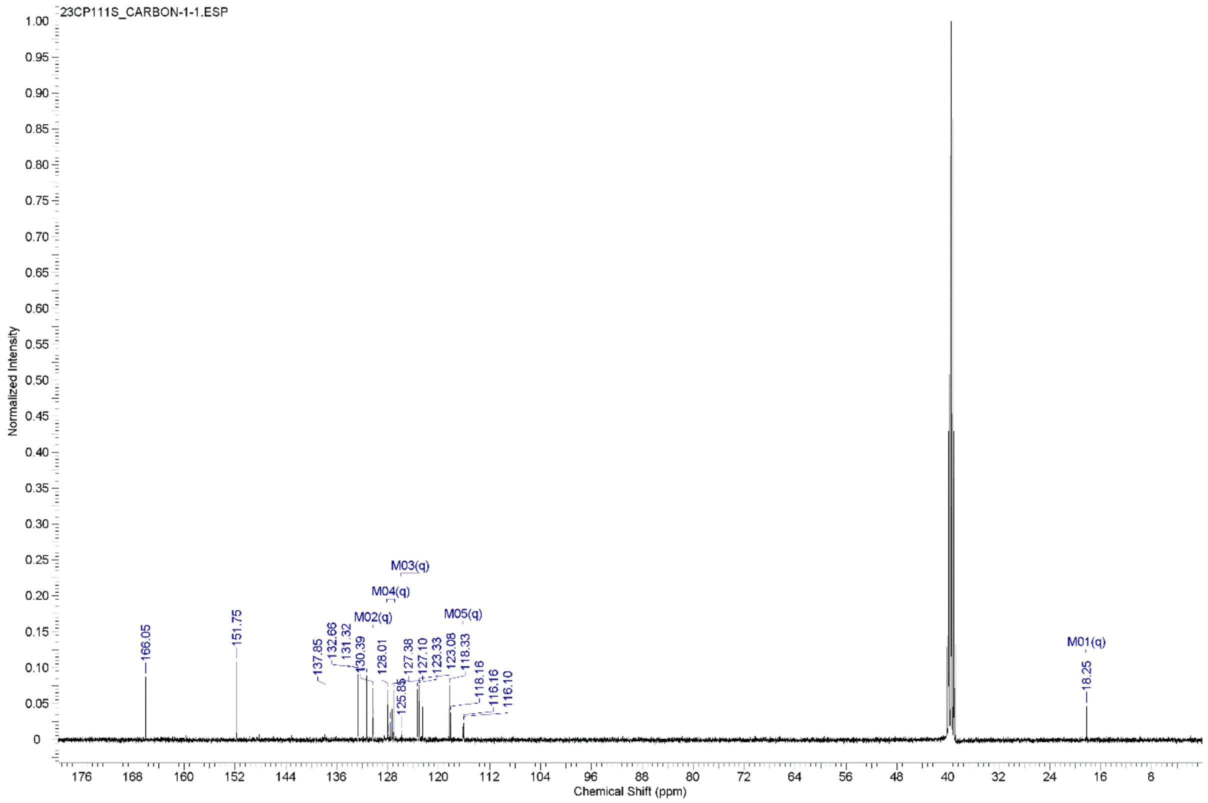

Figure S32. ^{13}C-NMR (DMSO-*d*$_6$) spectrum of 2-hydroxy-*N*-[4-methyl-3-(trifluoromethyl)phenyl]naphthalene-1-carboxamide (**23**)

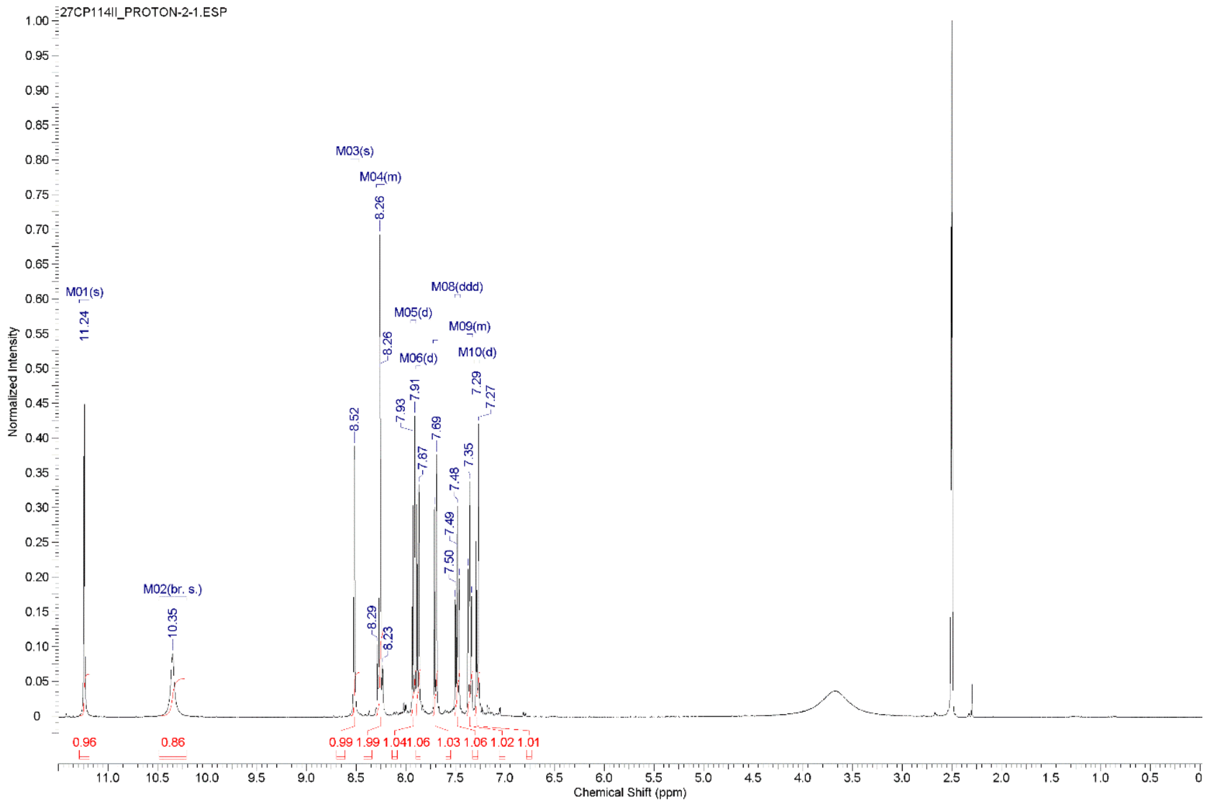

Figure S33. ^{1}H-NMR (DMSO-*d*$_6$) spectrum of 2-hydroxy-*N*-[4-nitro-3-(trifluoromethyl)phenyl]naphthalene-1-carboxamide (**27**)

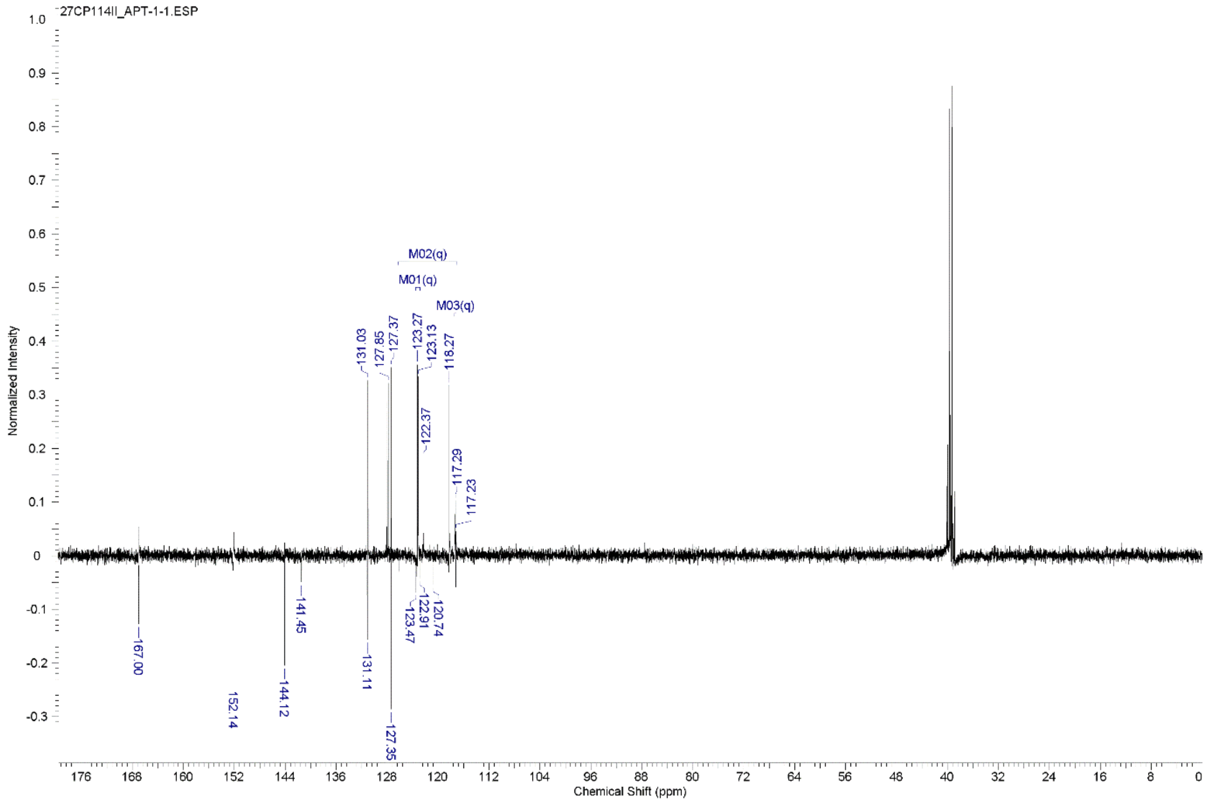

Figure S34. ^{13}C-NMR (DMSO-*d*$_6$) spectrum of 2-hydroxy-*N*-[4-nitro-3-(trifluoromethyl)phenyl]naphthalene-1-carboxamide (**27**)

CC BY
