# Supplementary material for: Antimicrobial and ADME properties of methoxylated, methylated and nitrated 2-hydroxynaphthalene-1 carboxanilides
Source: ADMET DMPK. 2025 Feb 8;13(1):2642. doi: 10.5599/admet.2642 (PMC11954145; doi:10.5599/admet.2642)
Supplement: Supplementary file 2 [file ADMET-13-2642-S1.docx]

ADMET & DMPK 13(1) (2025) S2642

*
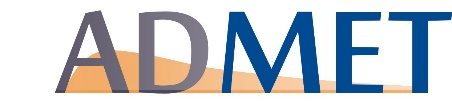
***Open Access : ISSN : 1848-7718**[***http://www.pub.iapchem.org/ojs/index.php/admet/index***](http://www.pub.iapchem.org/ojs/index.php/admet/index)

Supplementary material to

**Antimicrobial and ADME properties of methoxylated, methylated and nitrated 2-hydroxynaphthalene-1 carboxanilides**

Lucia Vrablova^1^, Tomas Gonec^2^, Tereza Kauerova^3^, Michal Oravec^4^, Izabela Jendrzejewska^5^, Peter Kollar^3^, Alois Cizek^6^ and Josef Jampilek^1,7^

^1^Department of Analytical Chemistry, Faculty of Natural Sciences, Comenius University, Ilkovicova 6, 84215 Bratislava, Slovakia
^2^Department of Chemical Drugs, Faculty of Pharmacy, Masaryk University, Palackeho tr. 1946/1, 61200 Brno, Czech Republic
^3^Department of Pharmacology and Toxicology, Faculty of Pharmacy, Masaryk University, Palackeho tr. 1946/1, 61200 Brno, Czech Republic

^4^Global Change Research Institute CAS, Belidla 986/4a, 60300 Brno, Czech Republic

^5^Institute of Chemistry, University of Silesia, Bankowa 12, 40007 Katowice, Poland

^6^Department of Infectious Diseases and Microbiology, Faculty of Veterinary Medicine, University of Veterinary Sciences Brno, Palackeho tr. 1946/1, 61242 Brno, Czech Republic
^7^Department of Chemical Biology, Faculty of Science, Palacky University Olomouc, Slechtitelu 27, 77900 Olomouc, Czech Republic

ADMET & DMPK **13(1)** (2025) 2642; <https://doi.org/10.5599/admet.2642>

**
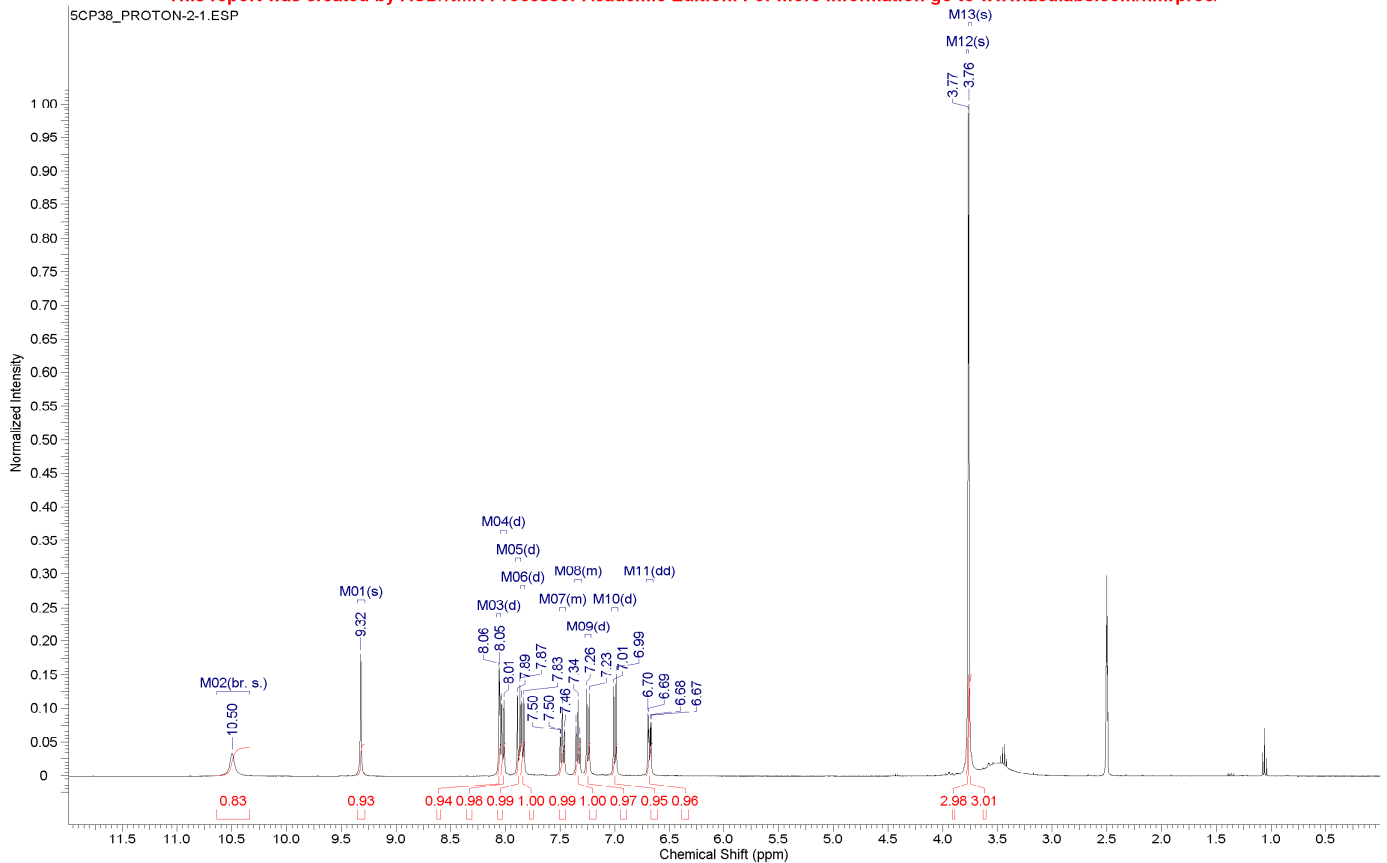
**

**Figure S1.** ^1^H-NMR (DMSO-*d_6_*) spectrum of
*N*-(2,5-dimethoxyphenyl)-2-hydroxynaphthalene-1-carboxamide (**5**)

**
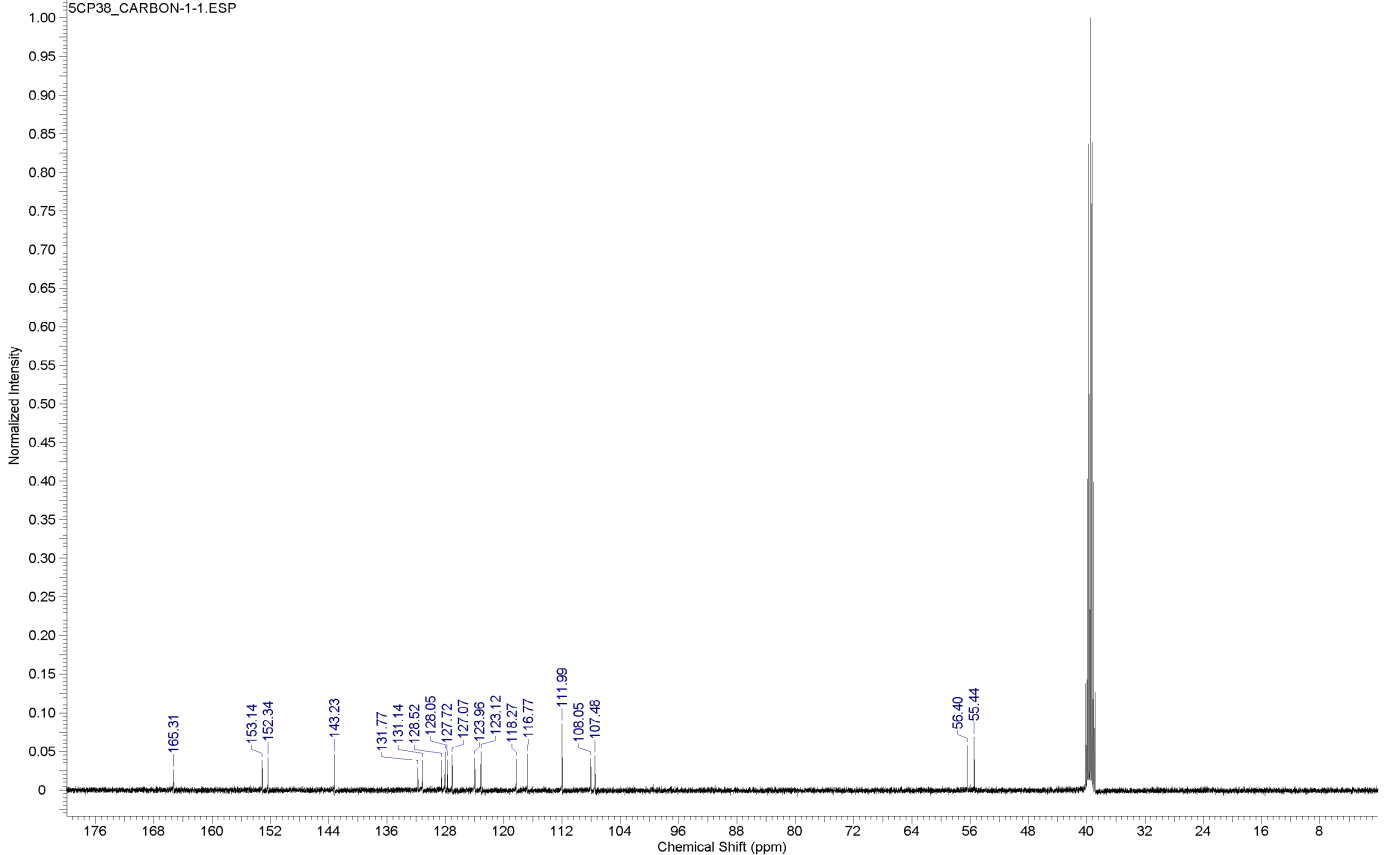
**

**Figure S2.** ^13^C-NMR (DMSO-*d_6_*) spectrum of
*N*-(2,5-dimethoxyphenyl)-2-hydroxynaphthalene-1-carboxamide (**5**)

**

**

**Figure S3.** ^1^H-NMR (DMSO-*d_6_*) spectrum of
*N*-(3,5-dimethoxyphenyl)-2-hydroxynaphthalene-1-carboxamide (**6**)





**Figure S4.** ^13^C-NMR (DMSO-*d_6_*) spectrum of
*N*-(3,5-dimethoxyphenyl)-2-hydroxynaphthalene-1-carboxamide (**6**)





**Figure S5.** ^1^H-NMR (DMSO-*d_6_*) spectrum of
2-hydroxy-*N*-(3,4,5-trimethoxyphenyl)naphthalene-1-carboxamide (**7**)





**Figure S6.** ^13^C-NMR (DMSO-*d_6_*) spectrum of
2-hydroxy-*N*-(3,4,5-trimethoxyphenyl)naphthalene-1-carboxamide (**7**)





**Figure S7.** ^1^H-NMR (DMSO-*d_6_*) spectrum of
*N*-(2,5-dimethylphenyl)-2-hydroxynaphthalene-1-carboxamide (**11**)





**Figure S8.** ^13^C-NMR (DMSO-*d_6_*) spectrum of
*N*-(2,5-dimethylphenyl)-2-hydroxynaphthalene-1-carboxamide (**11**)





**Figure S9.** ^1^H-NMR (DMSO-*d_6_*) spectrum of
*N*-(2,6-dimethylphenyl)-2-hydroxynaphthalene-1-carboxamide (**12**)





**Figure S10.** ^13^C-NMR (DMSO-*d_6_*) spectrum of
*N*-(2,6-dimethylphenyl)-2-hydroxynaphthalene-1-carboxamide (**12**)





**Figure S11.** ^1^H-NMR (DMSO-*d_6_*) spectrum of
*N*-(3,5-dimethylphenyl)-2-hydroxynaphthalene-1-carboxamide (**13**)





**Figure S12.** ^13^C-NMR (DMSO-*d_6_*) spectrum of
*N*-(3,5-dimethylphenyl)-2-hydroxynaphthalene-1-carboxamide (**13**)

**

**

**Figure** **S13**. ^1^H-NMR (DMSO-*d_6_*) spectrum of
2-hydroxy-*N*-(2,4,6-trimethylphenyl)naphthalene-1-carboxamide (**14**)





**Figure** **S14**. ^13^C-NMR (DMSO-*d_6_*) spectrum of
2-hydroxy-*N*-(2,4,6-trimethylphenyl)naphthalene-1-carboxamide (**14**)





**Figure S15.** ^1^H-NMR (DMSO-*d_6_*) spectrum of
2-hydroxy-*N*-(2-methoxy-5-methylphenyl)naphthalene-1-carboxamide (**15**)





**Figure S16.** ^13^C-NMR (DMSO-*d_6_*) spectrum of
2-hydroxy-*N*-(2-methoxy-5-methylphenyl)naphthalene-1-carboxamide (**15**)





**Figure S17.** ^1^H-NMR (DMSO-*d_6_*) spectrum of
2-hydroxy-*N*-(2-methoxy-6-methylphenyl)naphthalene-1-carboxamide (**16**)





**Figure S18.** ^13^C-NMR (DMSO-*d_6_*) spectrum of
2-hydroxy-*N*-(2-methoxy-6-methylphenyl)naphthalene-1-carboxamide (**16**)





**Figure S19.** ^1^H-NMR (DMSO-*d_6_*) spectrum of
2-hydroxy-*N*-(5-methoxy-2-methylphenyl)naphthalene-1-carboxamide (**17**)





**Figure S20.** ^13^C-NMR (DMSO-*d_6_*) spectrum of
2-hydroxy-*N*-(5-methoxy-2-methylphenyl)naphthalene-1-carboxamide (**17**)





**Figure S21.** ^1^H-NMR (DMSO-*d_6_*) spectrum of
*N*-(2-chloro-5-methoxyphenyl)-2-hydroxynaphthalene-1-carboxamide (**18**)





**Figure S22.** ^13^C-NMR (DMSO-*d_6_*) spectrum of
*N*-(2-chloro-5-methoxyphenyl)-2-hydroxynaphthalene-1-carboxamide (**18**)





**Figure S23.** ^1^H-NMR (DMSO-*d_6_*) spectrum of
*N*-(5-bromo-2-methoxyphenyl)-2-hydroxynaphthalene-1-carboxamide (**19**)





**Figure S24.** ^13^C-NMR (DMSO-*d_6_*) spectrum of
*N*-(5-bromo-2-methoxyphenyl)-2-hydroxynaphthalene-1-carboxamide (**19**)





**Figure S25.** ^1^H-NMR (DMSO-*d_6_*) spectrum of
2-hydroxy-*N*-[2-methoxy-5-(trifluoromethyl)phenyl]naphthalene-1-carboxamide (**20**)





**Figure S26.** ^13^C-NMR (DMSO-*d_6_*) spectrum of
2-hydroxy-*N*-[2-methoxy-5-(trifluoromethyl)phenyl]naphthalene-1-carboxamide (**20**)





**Figure S27**. ^1^H-NMR (DMSO-*d_6_*) spectrum of
2-hydroxy-*N*-[4-methoxy-3-(trifluoromethyl)phenyl]naphthalene-1-carboxamide (**21**).





**Figure S28**. ^13^C-NMR (DMSO-*d_6_*) spectrum of
2-hydroxy-*N*-[4-methoxy-3-(trifluoromethyl)phenyl]naphthalene-1-carboxamide (**21**)





**Figure S29.** ^1^H-NMR (DMSO-*d_6_*) spectrum of
2-hydroxy-*N*-[2-methyl-5-(trifluoromethyl)phenyl]naphthalene-1-carboxamide (**22**)





**Figure S30.** ^13^C-NMR (DMSO-*d_6_*) spectrum of
2-hydroxy-*N*-[2-methyl-5-(trifluoromethyl)phenyl]naphthalene-1-carboxamide (**22**)





**Figure S31.** ^1^H-NMR (DMSO-*d_6_*) spectrum of
2-hydroxy-*N*-[4-methyl-3-(trifluoromethyl)phenyl]naphthalene-1-carboxamide (**23**)





**Figure S32.** ^13^C-NMR (DMSO-*d_6_*) spectrum of
2-hydroxy-*N*-[4-methyl-3-(trifluoromethyl)phenyl]naphthalene-1-carboxamide (**23**)





**Figure S33.** ^1^H-NMR (DMSO-*d_6_*) spectrum of
2-hydroxy-*N*-[4-nitro-3-(trifluoromethyl)phenyl]naphthalene-1-carboxamide (**27**)





**Figure S34.** ^13^C-NMR (DMSO-*d_6_*) spectrum of
2-hydroxy-*N*-[4-nitro-3-(trifluoromethyl)phenyl]naphthalene-1-carboxamide (**27**)
